# A *LILRB1* variant with a decreased ability to phosphorylate SHP-1 leads to autoimmune diseases

**DOI:** 10.1038/s41598-022-19334-x

**Published:** 2022-09-14

**Authors:** Thivaratana Sinthuwiwat, Supranee Buranapraditkun, Wuttichart Kamolvisit, Siraprapa Tongkobpetch, Wanna Chetruengchai, Chalurmpon Srichomthong, Adjima Assawapitaksakul, Chureerat Phokaew, Patipark Kueanjinda, Tanapat Palaga, Tadech Boonpiyathad, Kanya Suphapeetiporn, Nattiya Hirankarn, Vorasuk Shotelersuk

**Affiliations:** 1 Interdisciplinary Program of Biomedical Sciences, Graduate School, Chulalongkorn University, Bangkok, Thailand; 2 Center of Excellence for Medical Genomics, Medical Genomics Cluster, Department of Pediatrics, Faculty of Medicine, Chulalongkorn University, Bangkok, 10330 Thailand; 3 Excellence Center for Genomics and Precision Medicine, King Chulalongkorn Memorial Hospital, the Thai Red Cross Society, Bangkok, Thailand; 4 Division of Cytogenetics, Chulabhorn Learning and Research Centre, Chulabhorn Royal Academy, Bangkok, Thailand; 5 Division of Allergy and Clinical Immunology, Department of Medicine, King Chulalongkorn Memorial Hospital, Faculty of Medicine, Chulalongkorn University, Thai Red Cross Society, Bangkok, 10330 Thailand; 6 Center of Excellence in Vaccine Research and Development (Chula Vaccine Research Center- Chula VRC), Faculty of Medicine, Chulalongkorn University, Bangkok, 10330 Thailand; 7 Thai Pediatric Gastroenterology, Hepatology and Immunology (TPGHAI) Research Unit, Faculty of Medicine, King Chulalongkorn Memorial Hospital, Chulalongkorn University, The Thai Red Cross Society, Bangkok, 10330 Thailand; 8 Research Affairs, Faculty of Medicine, Chulalongkorn University, Bangkok, 10330 Thailand; 9 Department of Microbiology, Faculty of Medicine, Chulalongkorn University, Bangkok, Thailand; 10 Center of Excellence in Immunology and Immune-Mediated Diseases, Chulalongkorn University, Bangkok, Thailand; 11 Department of Microbiology, Faculty of Science, Chulalongkorn University, Bangkok, Thailand; 12 Allergy and Clinical Immunology, Department of Medicine, Phramongkutklao Hospital, Bangkok, Thailand

**Keywords:** Computational biology and bioinformatics, Genetics, Immunology

## Abstract

Inborn errors of immunity are known to cause not only immunodeficiencies and allergies but also autoimmunity. Leukocyte immunoglobulin-like receptor B1 (LILRB1) is a receptor on leukocytes playing a role in regulating immune responses. No phenotypes have been reported to be caused by germline mutations in *LILRB1*. We aimed to identify the causative variant in a three-generation family with nine members suffering from one of the three autoimmune diseases—Graves’ disease, Hashimoto's thyroiditis, or systemic lupus erythematosus. Whole-genome linkage study revealed a locus on chromosome 19q13.4 with the maximum LOD score of 2.71. Whole-exome sequencing identified a heterozygous missense variant, c.479G > A (p. G160E) in *LILRB1*, located within the chromosomal-linked region, in all nine affected members. The variant has never been previously reported. Jurkat cells transfected with the mutant *LILRB1*, compared with those with the wild-type *LILRB1*, showed decreased phosphorylation of both LILRB1 and its downstream protein, SHP-1. Flow cytometry was used to study immunophenotype and revealed that LILRB1 was significantly lower on the surface of activated regulatory T lymphocytes (Treg) cells of patients. Single-cell RNA sequencing showed substantially increased M1-like monocytes in peripheral blood mononuclear cells of affected individuals. This study, for the first time, implicates *LILRB1* as a new disease gene for autoimmunity.

## Introduction

Inborn errors of immunity (IEIs) are heritable monogenic disorders affecting immune regulation and development^1^. IEIs have traditionally been associated with immunodeficiencies and susceptibility to infections; however, the presence of immune dysfunction also implicates them in autoimmunity and allergies. Although rare, IEIs provide biological insights into pathogenesis which paves the way for precision medicine and new therapeutic approaches.

Autoimmune diseases are the result of the failure of the immune system to develop tolerance toward self-antigens. They are characterized by the activity of autoreactive lymphocytes, which cause tissue or organ damage through the generation of antibodies that react against host tissues, or effector T cells, which are specific for endogenous self-peptides^2^. There is a wide range of autoimmune diseases whose manifestations depend mainly on the type of self-antigens that the immune system targets.

Hashimoto's thyroiditis (HT) and Graves' disease (GD) are autoimmune thyroid diseases in which the immune system generates autoantibodies against thyroglobulin and thyroperoxidase in HT and against thyrotropin receptor in GD, leading to the destruction of thyroid gland^3^. Systemic lupus erythematosus (SLE), mainly affecting the joints, skin, kidneys, brain, blood vessels, and serous membranes, is characterized by autoantibodies directed against nuclear antigens^4^.

Leukocyte immunoglobulin-like receptor B1 (LILRB1) is an inhibitory receptor, broadly expressing on many leukocytes, including NK cells, CD8+ and CD4+ lymphocytes, B lymphocytes, monocytes, and dendritic cells. The cytoplasmic region of LILRB1 contains four immunoreceptor tyrosine-based inhibition motifs (ITIMs). Upon tyrosine phosphorylation, ITIM recruits the Src homology 2 (SH2) domain-containing protein (SHP-1), which is a tyrosine phosphatase involved in the inhibition of different intracellular signal pathways^5^. Besides its role in adaptive immunity, LILRB1 can inhibit monocyte activation signals by dampening inflammatory signaling cascade^6^ and induces macrophage differentiation toward an M2 phenotype^7^. Moreover, LILRB1 has previously been shown to play an important role in immune response regulation^8^. Changes in its functions have been associated with autoimmune thyroid diseases^9^, SLE^5^, and other autoimmune diseases such as rheumatoid arthritis^10^.

Here, we identified a three-generation family with nine members affected by either GD, HT, or SLE. Whole-genome linkage (WGL) study and whole exome sequencing (WES) identified a variant in *LILRB1* in all nine patients. Functional studies demonstrated that the *LILRB1* variant had a decreased ability to phosphorylate SHP-1 indicated its pathogenicity. Flow cytometry and single-cell RNA sequencing (scRNA-seq) analyses suggested the decreased LILRB1 expression on Tregs and increased M1-like monocytes could participate in the disease pathogenesis. This study demonstrates that a germline loss-of-function variant in *LILRB1* could lead to autoimmune diseases.

## Results

### Clinical characteristics of autoimmune patients

**Figure 1:**
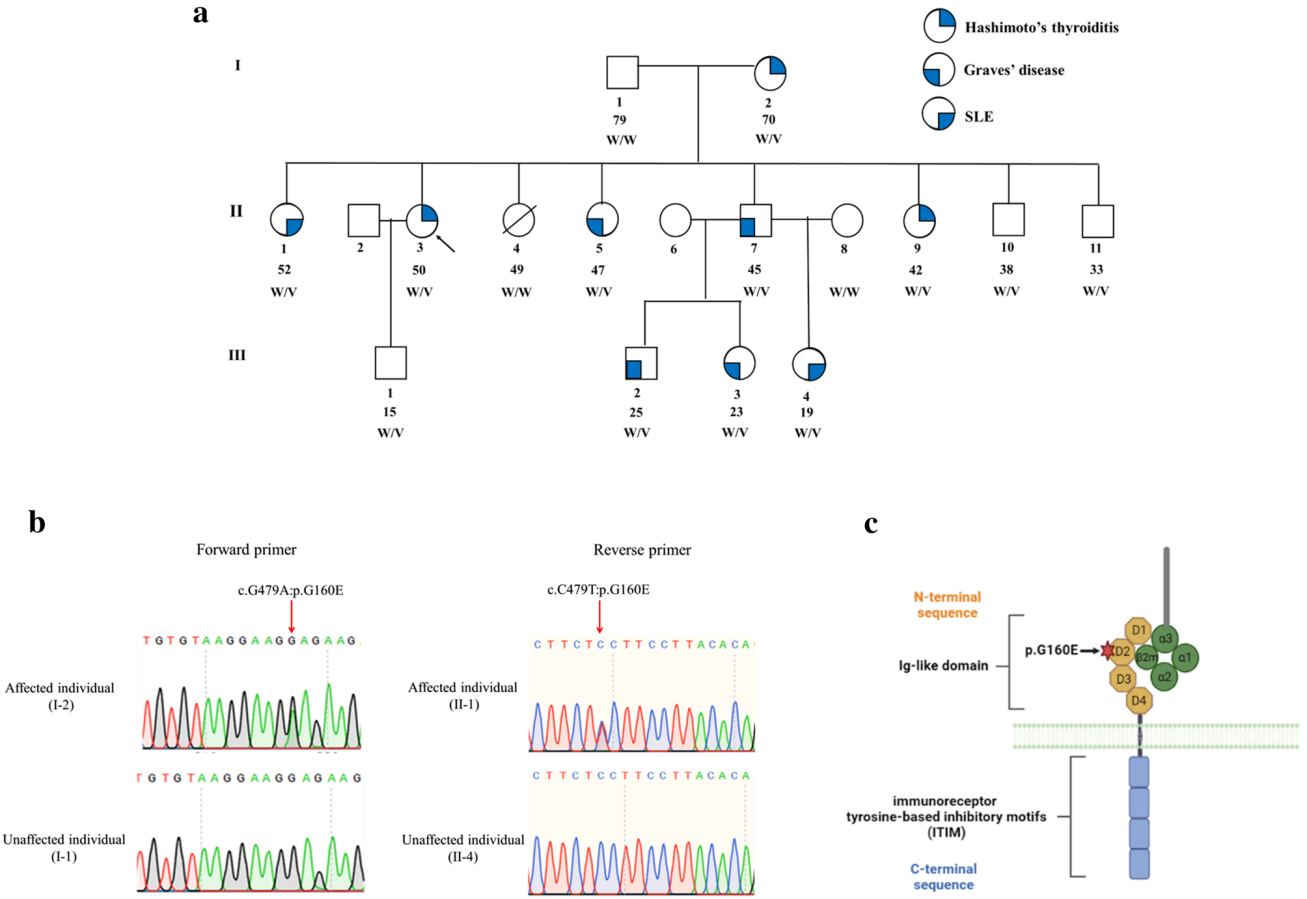
Whole-genome linkage analysis and exome sequencing identified a variant in *LILRB1* to be associated with familial autoimmune disease. (**a**) The family pedigree includes a symbolic presentation of the three clinical features of Hashimoto’s thyroiditis, Graves’ disease, and SLE. An arrow indicates the proband. The Roman numerals at the left side represent generations. Numbers immediately below individual’s symbols indicate individual’s order in the generation. Numbers below the individual’s order denote ages. W, wild-type allele. V, variant. (**b**) Sanger sequencing demonstrates a heterozygous missense c.479G > A (p.G160E) variant in *LILRB1*. (**c**) Structure of LILRB1 receptor. The arrow shows the p.G160E is located on the immunoglobulin-like domain (Ig-like C2-type 2).

Clinical and laboratory findings of the nine members suffering from one of the three autoimmune diseases—GD, HT, or SLE were characterized (Fig. 1a, Supplementary Table S1). The patient II-3 with HT and breast cancer was the proband who presented with a painless goiter and was referred by an oncologist to a clinical geneticist at King Chulalongkorn Memorial Hospital, Bangkok, Thailand. During pedigree taking, she was found to have several other family members with autoimmune diseases. All patients were seen at least once at King Chulalongkorn Memorial hospital, where history was reviewed, and a physical examination was performed. Laboratory tests were retrospectively reviewed from their primary hospitals including free T4, thyroid stimulating hormone (TSH), anti-thyroglobulin, anti-thyroid peroxidase, C-reactive protein, complete blood count, sedimentation rate, blood urea and creatinine concentrations, serum cholesterol and triglycerides, blood glucose test, 24-h urine protein test, urine analyses and renal biopsy result. Their clinical courses, immunological findings, and treatments were retrospectively reviewed.

### Whole-genome linkage (WGL) analysis and whole exome sequencing (WES) identified candidate genetic variants, and PCR-Sanger sequencing validated the variant’s existence

WGL analysis using the nine affected members defined the 4.5-Mb critical region on chromosomes 19q13.4 positions 53,861,258–58,379,941 (hg19) with a maximum logarithm of the odds (LOD) score of 2.71 (Supplementary Fig. S1a-1c). WES with filtering steps (Supplementary Table S2) revealed three non-synonymous exonic variants present in all nine patients with allele frequencies < 1% in public and in-house databases^11^ (Supplementary Table S3). Remarkably, only one variant was located on the chromosomal linked region, which was a heterozygous missense mutation in *LILRB1* (hg19; chr19:55,143,506; c.479G > A; p.Gly160Glu; rs866926837). Its allele frequency in the East Asian population in the gnomAD database (https://gnomad.broadinstitute.org) is 0.0001503 but not in the ClinVar and the 1000 Genomes Project databases. Germline variants including the c.479G > A in *LILRB1* have never been previously reported to cause any diseases. Thus, we used PCR-Sanger sequencing to confirm its presence in all nine patients (Fig. 1b). The p.G160E is located on the immunoglobulin-like C2-type 2 domain^12^, an important region for binding to a major histocompatibility complex^10^ (Fig. 1c). To confirm the important of this amino acid residue, we compared the amino acid sequences of this region across multiple species, ranging from human to *C. elegans*, and found that this amino acid was evolutionarily conserved (Supplementary Fig. S1d).

### Quantitative real-time PCR and Western blot analysis showed the LILRB1 variant did not affect RNA and protein expressions

**Figure 2:**
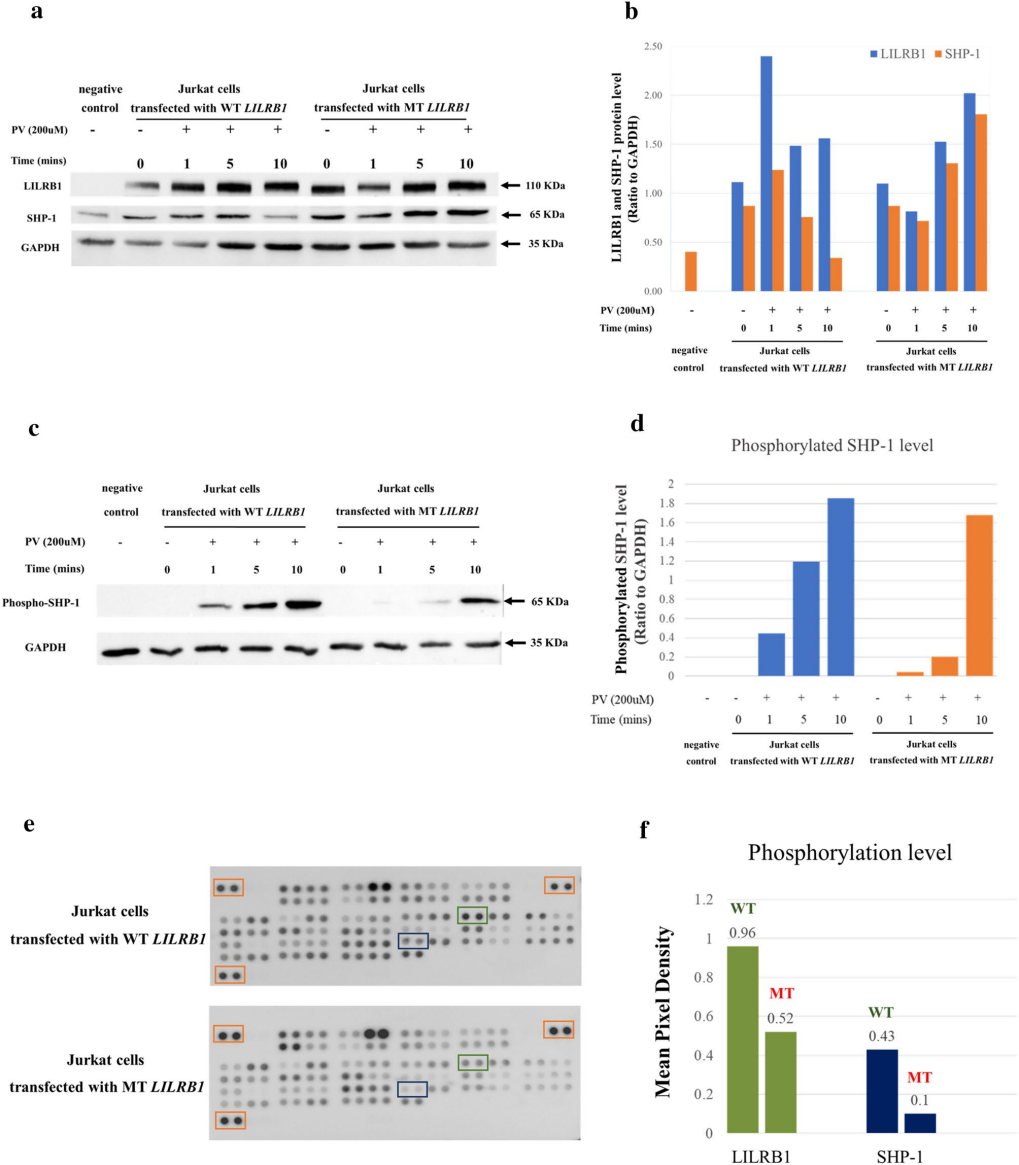
The c.479G > A (p.G160E) variant in LILRB1 decreases SHP-1 phosphorylation. (**a**) The protein levels of LILRB1 and SHP-1 in the Jurkat cells transfected with the wild-type (WT) *LILRB1* and mutant (MT) *LILRB1* in different time points as detected by Western blotting. GAPDH was used as a loading control. The grouping of blots was cropped from different parts of the same gel. Control denotes Jurkat cells without transfection and PV denotes pervanadate.(**b**) Representative Western blot graphs showing relative densitometric bar graphs of total proteins of LILRB1 and SHP-1 normalized to the intensity of the corresponding GAPDH bands. (**c**) The tyrosine phosphorylation status of SHP-1 was examined using Western blot analysis. The grouping of blots was cropped from different parts of the same gel. (**d**) Representative western blot graphs showing relative densitometric bar graphs of phosphorylated SHP-1 and GAPDH. (**e**) Representative blots of Human Phospho-Immunoreceptor Antibody Arrays treated with PV and incubated with a phosphotyrosine-specific antibody. Dots of LILRB1, SHP-1, and controls are boxed in green, blue, and orange, respectively. (**f**) Phosphorylation levels of the dot blots. The relative change in the phosphorylation state of LILRB1 and SHP-1 (the average signal of the pixel density of the pair of duplicate spots) to the average of signal reference spots in three corners of the array. The original Western blots are presented in Supplementary Fig. S8 and S9, respectively.

To elucidate the effect of this mutation on LILRB1 function, we first measured mRNA expression level of this gene in peripheral blood mononuclear cells (PBMCs) by quantitative real-time PCR (qRT-PCR). The result showed no significant difference of *LILRB1* mRNA levels among the unaffected members without the *LILRB1* variant, the unaffected with the variant, and the affected with the variant (Supplementary Fig. S2). Western blot analysis detected a similar amount of LILRB1 protein was found in Jurkat cells transfected with the wild-type (WT) *LILRB1* compared to those transfected with the mutant (MT) *LILRB1* (Fig. 2a,b). These results suggested that the c.479G > A in *LILRB1* did not affect RNA and protein expressions.

### Western blot analysis and human phospho-immuno receptor antibody array found decreased phosphorylation levels of both LILRB1 and SHP-1 in the mutant LILRB1

To verify that p.G160E in *LILRB1* was a loss-of-function variant, we compared the LILRB1 and SHP-1 phosphorylation levels in Jurkat cells transfected with either WT or MT *LILRB1* in the presence of pervanadate (PV)—a substance inducing tyrosine phosphorylation of LILRB1^13^. Western blot analysis revealed decreased phosphorylation levels of SHP-1 in Jurkat cells transfected with MT *LILRB1*, compared with those transfected with WT one (Fig. 2c,d). In addition, immunoblot using antibody array of human phospho-proteins at 10 min showed decreased phosphorylation levels of both LILRB1 and SHP-1 (Fig. 2e,f). These findings indicated that p.G160E mutation impaired LILRB1 phosphorylation ability, which in turn reduced SHP-1 phosphorylation, implying that this variant in *LILRB1* gene caused a loss-of-function.

### Flow cytometry analysis showed the surface LILRB1 expression in PBMC of patients

**Figure 3:**
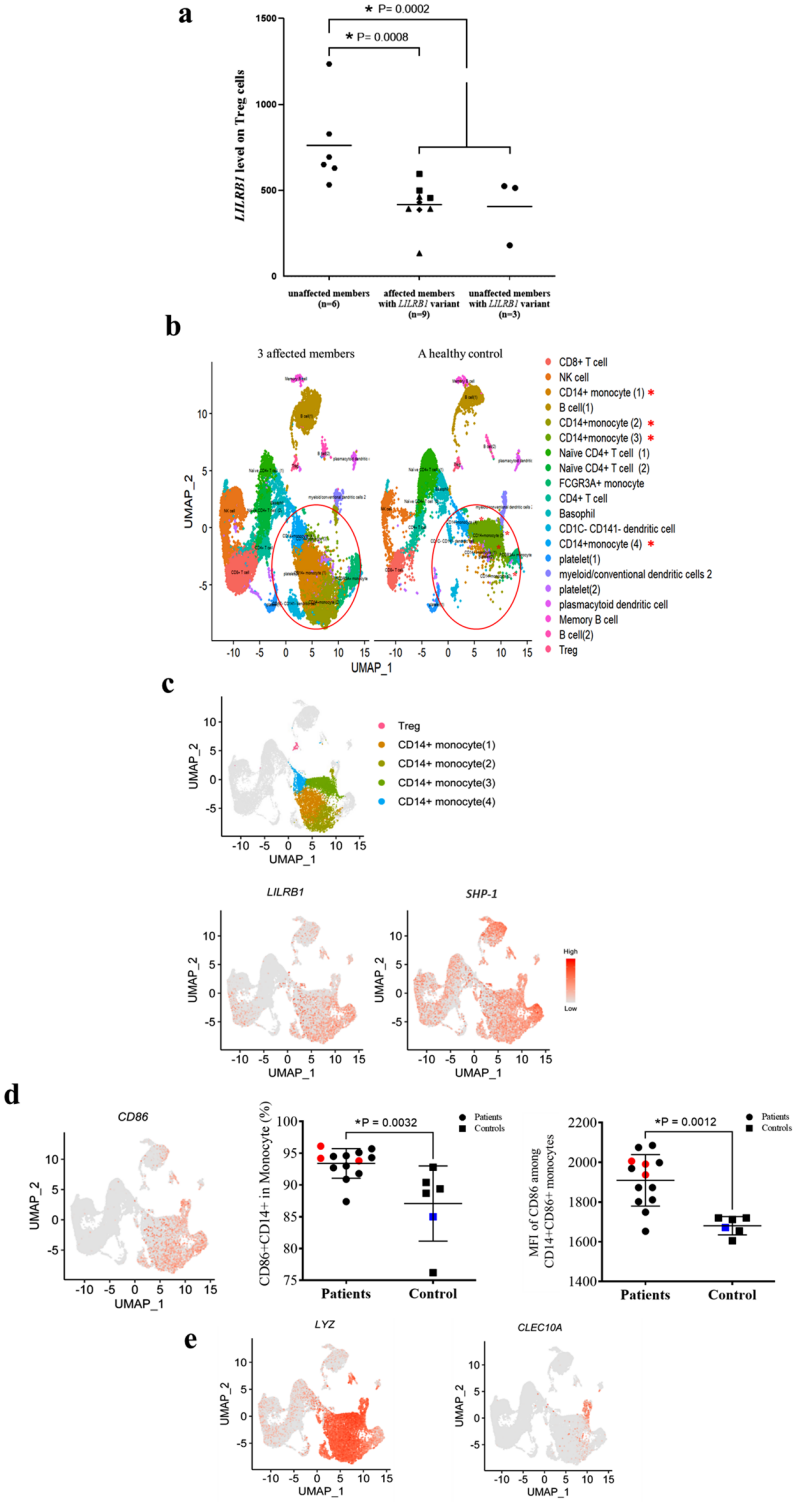
Flow cytometry and single cell RNA sequencing (scRNA-seq) results. (a) Comparison of mean fluorescent intensity (MFI) of LILRB1 expression on the surface of activated Treg cells in family members (**b**) High-dimensional transcriptomic scRNA-seq clustering reveals increased percentage of monocyte subsets in the three studied patients compared with the sex-, age- and ethnic-matched control. (**c**) Two-dimensional UMAP visualization of PBMCs of patients with the *LILRB1* variant. Colors represent four clusters (cell types) of interest similar to (**b)** (top panel). Expression of marker genes for *LILRB1* and *SHP-1* (bottom panel). Red color indicates high expression level of the gene. **(d)** UMAP visualization of PBMCs of patients with the macrophage subset markers *CD86* (left panel) and flow cytometry results of the frequency (middle panel) and MFI (right) of CD86 marker, where red and blue dots indicate samples of patients and controls concurrently investigated in scRNA-seq experiment, respectively. * indicates statistical significance. **(e)** *LYZ* (left panel) and *CLEC10A* (right panel) expression in cell population**.** Visualization of single-cell transcriptome data was done in R (v. 4.2.1; https://www.R-project.org) using RStudio (http://www.rstudio.com) and R packages tidyverse (v. 1.3.1; https://doi.org/10.21105/joss.01686) and ggpubr (v. 0.4.0; https://CRAN.R-project.org/package=ggpubr).

After the loss-of-function of the variant was substantiated, we further sought to find abnormalities in patients’ PBMCs that might suggest disease pathogenesis. Flow cytometry analysis revealed that the surface LILRB1 expression on activated regulatory Treg (GARP+LAP+CD25+CD127-CD4+) cells was significantly decreased in the family members with the *LILRB1* variant. According to the flow cytometry data, the surface LILRB1 expression level on the activated regulatory Tregs in symptomatic (median = 431) or asymptomatic (median = 444) patients was significantly lower than in those without the variant (median = 673) (difference of median = -242; P = 0.0008 and difference of median = -229; P = 0.0002 for symptomatic vs. control and asymptomatic vs. control, respectively) (Fig. 3a).

We also analyzed the expression of LILRB1 in different leukocyte populations and found no differences in the percentage of LILRB1 expressing CD4+ and CD8+ T lymphocytes, NK cells (CD16+CD56+), B cells (CD19+), monocytes (CD14+), myeloid dendritic cells (mDCs; CD1c+CD11c+), plasmacytoid dendritic cells (pDCs; CD123+CD303+) and regulatory B lymphocytes (Breg; CD71+CD73-CD25+CD19+) between the patients with the *LILRB1* variant and the controls (Supplementary Fig. S3a–S3g). Notably, this is the first time LILRB1 expression level was observed in Breg cell populations.

### Single-cell RNA-seq analysis revealed expansion of CD14+ monocyte subpopulations

We further performed scRNA-seq analysis of PBMCs from three patients and a healthy sex-, age- and ethnic-matched control to further characterize leukocyte population. Notably, the result showed a substantial expansion of CD14+ monocyte population in patient sample compared to healthy control (Fig. 3b and Supplementary Table S4). Among the CD14+ monocytes, four subsets of unique monocytes were identified. Remarkably, the highest increase was CD14+ monocyte 2 subset (1,063 cells per patient compared to 35 cells in the control; log_2_ fold-change = 4.9) while the most decrease was CD14+ monocyte 3 subset (35 cells per patient compared to 3,039 cells in the control; log_2_ fold-change = -6.5). Moreover, scRNA-seq result showed an increase in *LILRB1* and *SHP-1* expression levels in CD14+ monocyte subsets (Fig. 3c).

In addition, we annotated the CD14+ monocytes to macrophage subsets using MacSpectrum^14^, a 2-index platform that allows mapping of macrophage activation states. The analysis suggested that CD14+ monocyte 2 and 3 subsets had M1- and M2-like phenotypes, respectively (Supplementary Fig. S4). LILRB1 was previously found to polarize monocytes toward M2-like macrophages^15^. Consistently, CD14+ monocyte subsets in our patients who harbored the loss-of-function *LILRB1* variant substantially expressed *CD86* (a maker of mature macrophage) (Fig. 3d, left panel) and *LYZ* (M1 marker) but not *CLEC10A* (M2 marker) (Fig. 3e), indicating a highly active pro-inflammatory M1-like CD14+ monocyte population in these patients.

### Flow cytometry showed increased total monocytes and M1 monocytes

Next, we used flow cytometry to confirm our findings in the scRNA-seq results by staining PBMCs from the three patients and the controls with antibodies against the M1 or M2 signatures. The gating strategy of M1 and M2 monocytes is depicted in Supplementary Fig. S5.

We found an increasing tendency of the frequency of CD14+ monocytes in patients (median = 24.30) when compared to healthy controls (median = 16.75) (difference = 7.55; P = 0.0577; Supplementary Fig. S6a). When determining the M1 monocytes using CD80 and CD86 markers, we found that the frequency of M1 (CD14+CD80+CD86+) monocytes in patients and controls were not different (Supplementary Fig. S6b). However, when considering CD14+CD80+ monocytes and CD14+CD86+ monocytes separately, we found that the frequency (-5.15; P = 0.0032) and MFI (-245; P = 0.0012) of CD14+CD86+ monocytes significantly increased in patients (Fig. 3d; middle and right panels) but not CD14+CD80+ monocytes (Supplementary Fig. S6c-S6d). The increase of CD86 frequency and MFI in CD14+ monocytes from patients revealed by flow cytometry supported the results of our scRNA-seq analysis, which indicated a higher CD86 expression in CD14+ monocyte population from the patients.

In addition to the M1-like monocyte population, we examined the M2-like monocytes using the M2 markers, CD163 and CD206. We found that the frequency of M2 (CD14+CD163+CD206+) monocytes in patients was not statistically different from that in healthy controls (0.00343; P = 0.0573; Supplementary Fig. S6e). The analyses of CD14+CD163+ monocytes and CD14+CD206+ monocytes revealed that the frequency and MFI of both CD163 and CD206 in patients were not different from those in healthy controls (Supplementary Fig. S6f.-S6i). From our findings, we hypothesized that the loss-of-function mutation of *LILRB1* might increase a tendency of M1 polarization and caused M1/M2 ratio imbalance. Thus, we calculated M1/M2 ratio by dividing the total number of M1 macrophages by the total number of M2 macrophages in each patient and healthy group. The M1/M2 ratio in patients (ratio = 35.97) was significantly higher than in healthy controls (ratio = 17.68) (difference = 18.29; P = 0.1061.) (Supplementary Fig. S6j). The loss-of-function mutation of *LILRB1* was implicated in a skewing of monocytes toward an M1 polarization, resulting in an imbalance of M1/M2 ratio.

### Single-cell RNA-seq analysis of five additional individuals showed increased CD14+ monocyte subpopulations

To further validate our findings from scRNA-seq analysis, we incorporated publicly available scRNA-seq data of PBMCs from five healthy females aged 25–29 years old into our scRNA-seq dataset (Supplementary Table S5). Although the cell topology was not preserved as a result of the addition of the new samples into the dataset (Supplementary Fig. S7), we attempted to maintain consistency in cell-type classification by applying CIBERSORTx^16–18^ to identify cell types based on the gene expression profiles used in our previous scRNA-seq analysis (Supplementary Table S6).

The scRNA-seq analysis revealed that the total number of CD14+ monocytes in patient samples was more than that of healthy controls (1,580 cells per patient compared to 536 cells in a control; log_2_Fold-change = 1.56) (Supplementary Table S7). The number of the CD14+ monocyte subset expressing *LYZ* (clusters 2 and 4) representing M1-like cells was more prevalent in patients than in healthy controls. In contrast, the CD14+ monocyte subset expressing *CLEC10A* representing M2-like cells was absent in both patients and healthy controls. The increased numbers of total CD14+ monocytes and M1-like cells were consistent with our flow cytometry results.

## Discussion

Here, we identified a three-generation family with nine members suffering from one of the three autoimmune diseases – Grave’s disease, Hashimoto’s thyroiditis, or SLE. Three family members (individuals #II-10, II-11, III-1 in Fig. 1a) who had been healthy were found to harbor a *LILRB1* variant. Notably, all three were male with relatively young age. As it remains possible that they could develop symptoms or be affected when they become older, a regular follow-up visit would be prudent. Of the 12 members who harbor the variant, nine are affected, suggesting a 75% penetrance.

Previous studies showed that phosphorylated LILRB1 recruits and phosphorylates SHP-1, which inhibits both signaling and cellular events important for T cell activation^13^. Since the identified p.G160E is located in the Ig-like C2-type 2 domain of LILRB1—an important binding region to its cognate ligand such as MHC-I^19^, we hypothesized that this *LILRB1* variant resulted in a loss-of-function; in the presence of its ligand, the mutant LILRB1 would have a decreased phosphorylation of itself and its downstream molecule, SHP-1. This might lead to an over-activation of some T cell clones and subsequently cause autoimmunity. The study of p. G160E showed that phosphorylation of *LILRB1* and *SHP-1* decreased when compared with wild-type. These indicate that it possesses a loss-of-function mechanism, in the presence of its ligand.

Because p.G160E is a germline mutation, we profiled leukocytes in PBMCs of the patients by flow cytometry and found that surface LILRB1 expression on activated Treg cells was significantly decreased in the family members with the *LILRB1* variant, either symptomatic or asymptomatic, compared with those without the variant. Tregs are immunosuppressive cells that play a crucial role in regulating immune tolerance in pathological settings and in preventing autoimmune diseases. Treg deficiency, reduction, and dysfunction all account for various autoimmune diseases^20^. The dysfunction of Tregs is one of the proposed mechanisms underlying the breakdown of self-tolerance, leading to the progression of autoimmunity. The binding of HLA-G to LILRB1 on NK cells, T cells, and macrophages can inhibit the cytotoxicity of NK cells and CD8+ T cells as well as increase the number Treg cells, contributing to development of immune tolerance^21^. We hypothesize that the loss-of-function variant in *LILRB1* with a significantly lower LILRB1 on the surface of activated Treg cells might lead to defective suppressive function and immune regulation failure leading to autoimmune diseases^22^.

Single-cell RNA-seq analysis was often used to identify immune cell subsets related to the disease^23^. In addition to Treg cells, our scRNA-seq analysis revealed an expansion of CD14+ monocyte population in patients. Monocytes have been described for their broad immuno-modulatory, inflammatory, and tissue-repairing roles in development of autoimmune diseases. In particular, after getting exposure to specific cytokines in the tissue environment, naïve monocytes can differentiate into macrophages having either pro-inflammatory or anti-inflammatory functions, known as M1- or M2-like macrophages, respectively^24^. Furthermore, the pathogenesis of autoimmune diseases can be manifested as an imbalance between pro-inflammatory M1- and wound healing M2-like macrophages^25^. Based on our scRNA-seq data, we determined that CD14+ monocyte 2 and 3 subsets were M1- and M2-like cells when we used *LYZ* and *CLEC10A* to define M1- and M2-like monocytes, respectively^18,26,27^. In addition to scRNA-seq results, we identified the M1 and M2 monocyte populations in PBMCs of patients were examined by flow cytometry using typical M1 (CD80 and CD86) and M2 (CD163 and CD206) markers. A significant increase in frequency and MFI of CD86+ monocyte subset was observed in patients. These results strongly imply that the *LILRB1* variant investigated here affects the phenotypes of monocytes by biasing toward M1-like phenotype. It remains unclear whether the observed impact on monocytes is intrinsic or extrinsic and warrants for further investigation. This increased *LILRB1* and *SHP-1* expression in patients’ monocytes might indicate a compensatory mechanism for the loss of LILRB1 functions.

Despite a change in cell topology after addition of more data (five healthy female individuals) into our scRNA-seq dataset, our conclusion regarding the CD14+ monocyte 1 subset as M1-like cells that could contribute to the observed inflammation in patients remains intact as we mentioned that the loss-of-function mutation of *LILRB1* resulted in the tendency toward an M1 polarization and M1/M2 ratio imbalance.

**Figure 4:**
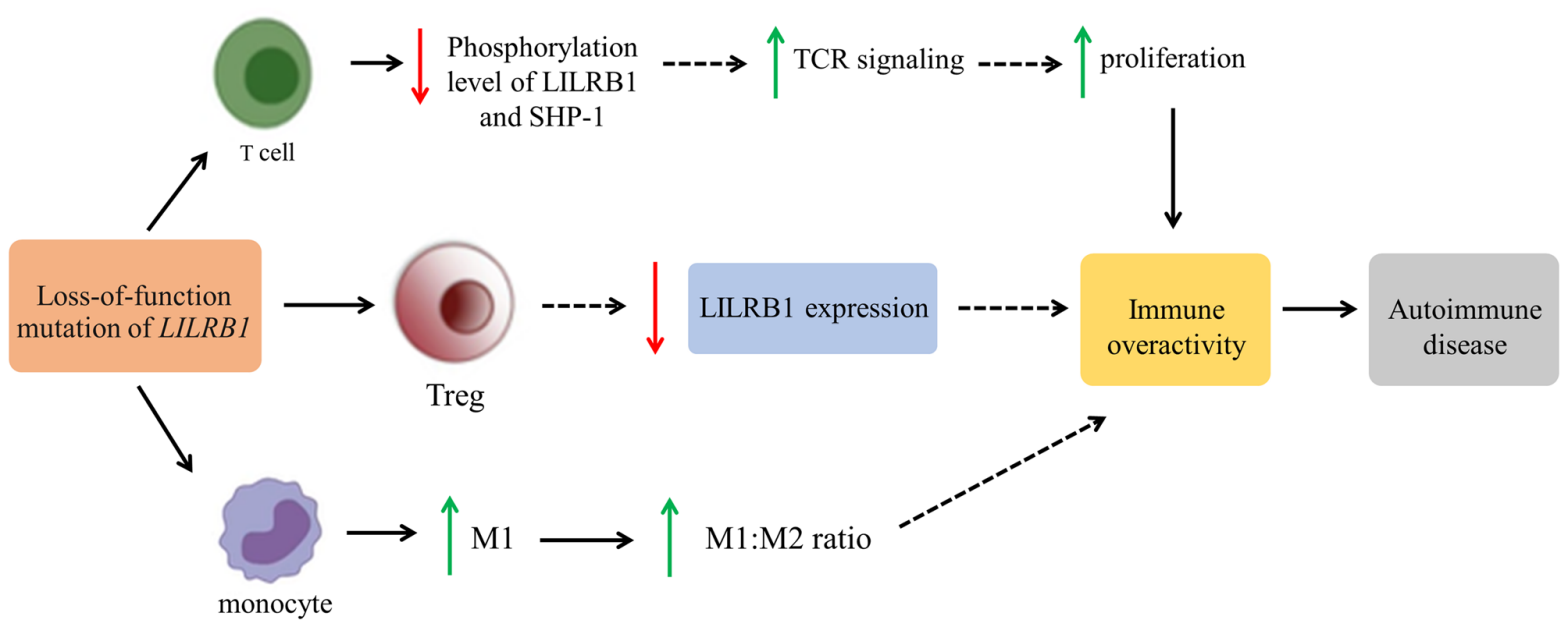
Proposed diagram of the loss-of-function *LILRB1* variant resulting in immune over activity and autoimmune diseases.

In summary, this study is the first to implicate *LILRB1* as a new monogenic disease gene for autoimmunity. Loss-of-function *LILRB1* variant decreases the ability to phosphorylate SHP-1, involves in reduced LILRB1 expression on the surface of activated Treg cells, and caused an imbalance in the M1/M2 macrophage ratio. This may result in a break of immune tolerance and hyperactivation of pro-inflammatory immune cells, subsequently leading to the development of autoimmune diseases (Fig. 4).

## Methods

### Subject recruitment

A three-generation family with nine members suffering from one of the three autoimmune diseases – Grave’s disease, Hashimoto’s thyroiditis, or SLE that fulfilled the diagnostic criteria, was recruited. All experimental protocols were approved by the institutional review board of the Faculty of Medicine, Chulalongkorn University (IRB #360/61), and all methods were performed under the guidelines and regulations mandated by the board. Written informed consent was obtained from all enrolled subjects, and their peripheral blood samples were collected.

### Whole-genome linkage analysis (WGL)

Nine members of the family (I-2, II-1, II-3, II-5, II-7, II-9, III-2, III-3 and III-4) were genotyped using Infinium OmniZhongHua-8 BeadChip specific to Chinese populations containing 1,175,489 single-nucleotide polymorphisms (SNPs; Illumina, San Diego, CA, USA). Parametric linkage analysis showed LOD score calculation for a phase-known data (Fig. S1a) and was performed by Merlin 1.1.2 software using an autosomal dominant model with the penetrance values being set at 0.6 (Fig. S1b-S1c).

### Whole-exome sequencing (WES)

WES was performed as previously described^28^. Briefly, genomic DNA was isolated from peripheral blood mononuclear cells using an extraction kit (Qiagen Inc., Valencia, CA, USA). The DNA sample was prepared as an Illumina sequencing library and in the exome capture step. The sequencing libraries were enriched by SureSelect Human All Exon V7 kits. The captured libraries were sequenced using Illumina NovaSeq 6000 Sequencer. The sequences were aligned to the human genome reference sequence (UCSC Genome Browser, hg19). To identify disease-causing variants under the assumption of an autosomal dominant pattern of inheritance. The analysis was made and filtered all SNVs and Indels; located in exons or flanking introns of the listed genes and not synonymous (Supplementary Table S2). The identified variants were validated using Sanger sequencing.

### Primers for PCR amplification and sequencing

To confirm the presence of the identified variant, we performed PCR and Sanger sequencing in the affected and unaffected family members who underwent WES. The primers amplified DNA and the PCR products were treated with Exo-SAP-IT (Affymetrix), followed by Sanger sequencing. The primer sequences are shown in Supplementary Table S8^10^.

### Quantitative real-time PCR

We performed quantitative real-time PCR (qRT-PCR) with RNA from PBMCs of eight patients from the family (II-1, II-3, II-5, II-7, II-9, III-2, III-3 and III-4), using the TaqMan® Gene Expression Assay (Applied Biosystems, cat # APT2AFH for LILRB1, Hs01060665_g1 ACTB, respectively). The *LILRB1* expression levels were calculated relative to the reference gene, *ACTB*. These were compared with two unaffected controls, who were unaffected family members.

### Mutagenesis

The expression vectors pcDNA3.1+ /C-(K)DYK containing the wild-type *LILRB1* were purchased from GenScript. The mutant *LILRB1* vectors of p.G160E was generated using Q5 Site-Directed Mutagenesis Kit (New England Biolabs, Ipswich, MA, USA). After mutagenesis, the mutant plasmid was extracted, and the sequence was verified by Sanger sequencing.

### Cells and cell transfection

T lymphocyte cell line, Jurkat, were grown in RPMI1640/10% FBS. The wild-type and mutant *LILRB1* plasmids were transfected to Jurkat cells (without endogenous LILRB1 expression) by electroporation using AmaxaTM SE Cell Line 4D-NucleofectorTM X Kit S a Lonza 4d strip nucleocuvette (Lonza, Germany). The transfectants were selected in G418-containing medium. LILRB1 expression on transfected cells was assessed by FACS analysis using anti-LILRB1 mAb GHI/75 (BioLegend, San Diego, CA, USA).

### Western blot analysis

Total proteins from Jurkat cells treated with pervanadate (PV) (200 μM sodium orthovanadate and 200 μM H_2_O_2_ at 37 °C for 5 and 10 min) were lysed in RIPA buffer (Thermo Fisher Scientific). Total amount of protein was measured by the PierceTM BCA Protein Assay Kit (Thermo Scientific, Rockford, IL, USA). The results of three independent experiments were reported as mean ± SD. The P-value was < 0.01. A total amount of 20 μg of protein per sample was separated on 12% sodium dodecyl sulfate–polyacrylamide gels and then transferred by iBlot™ Transfer Stack onto regular-size PVDF membrane (Invitrogen, Carlsbad, CA, USA). Monoclonal anti-LILRB1 antibody at 1:500 dilution (cat # 78144s, Cell Signaling Technology, Danvers, MA, USA) was used as primary antibody to detect WT and MT LILRB1 proteins followed by anti-rabbit IgG antibody at 1:2000 dilution (cat # 7076, Cell Signaling Technology) as secondary antibody. Anti-GAPDH antibody (cat # Mab1501, Sigma-Aldrich, St Louis, MO, USA) was used as a positive control to determine gel loading equivalency. Monoclonal Phospho-SHP-1 (Tyr564) (D11G5) antibody at 1:1000 dilution (cat # 8849, Cell Signaling Technology) was used as primary antibody to detect phospho-SHP1 proteins. The results were visualized using SuperSignal™ West Pico PLUS Chemiluminescent Substrate (Thermo fisher scientific) and chemiluminescence camera (ImageQuant LAS 4000, Amersham). The level of the proteins were quantified using the Image J gel analysis program.

### Human phospho-immunoreceptor array

Jurkat cells transfected with *LILRB1*-WT and *LILRB1*-MT expression vectors followed by pervanadate (PV) treatment were lysed as described above. A total of 20 μg of protein per sample was used to determine tyrosine phosphorylation level of LILRB1 and SHP-1 by Human Phospho-Immunoreceptor Array (R&D Systems, cat # ARY004) according to the manufacturer’s instructions. Moreover, the phosphorylation level of SHP-1 was also determined by Western blot analysis and quantified using the Image J gel analysis program.

### Flow cytometry to determine expression of LILRB1 from PBMCs

To evaluate the expression of LILRB1 in different cell populations from the PBMCs of 14 family members and five healthy controls. PBMCs were stained with specific primary antibodies. For T B NK panel, anti-CD4 APC-Cy7 (clone RPA-T4), anti-CD8 AF700 (clone SK1), anti-CD14 PE-Cy7 (clone HCD14), anti-CD16 PE-DZ594 (clone 3G8), anti-CD19 PE-Cy5 (clone HIB19), anti-CD56 AF647 (clone 5.1H11) were used. For DC panel, anti-CD1C PE-DZ594 (clone L161), anti-CD11c AF700 (clone N418), anti-CD123 APC (clone 6H6), and anti-CD303 PerCP-Cy5.5 (clone 201A) were used. For the Treg panel, anti-CD25 APC-Cy7 (clone BC96), anti- CD127 PE-DZ594 (clone A019D5), anti-GARP PE-Cy7 (clone 7B11), and anti-LAP APC (clone TW4-2F8) were used. For the Breg panel, anti-CD19 PerCP-Cy5.5 (clone HIB19), anti-CD24 PE-DZ594 (clone ML5), anti-CD38 AF700 (clone HB7), anti- CD71 PE-Cy7 (clone CY1G4), and anti-CD73 APC (clone AD2) were used. In addition, an anti-LILRB1 MAB (clone HP-F1) labeled with phycoerythrin (PE, Invitrogen) was also employed.

### Single-cell RNA sequencing (scRNA-Seq)

#### Cell isolation, capturing, library preparation and sequencing.

PBMCs were isolated using density-gradient centrifugation from heparinized peripheral blood. PBMC were stored at − 80 °C in the freezing medium for use. All samples were washed and resuspended in PBS, containing 0.1% BSA. Cell numbers and cell viability for each sample were counted using an automated cell counter (Countess II, Invitrogen) before single-cell RNA-seq library preparation. The cell numbers of three patients were 16,486, 16,454, and 16,517 cells whereas the cell number of a healthy control was 16,511 cells. The viability of cells in all samples was greater 80%.

According to the manufacturer's protocol^29^, the single-cell capturing and downstream library constructions were performed using the Chromium Single Cell 5′ library or 3' v2 library preparation kit (10X Genomics). Briefly, cellular suspensions were partitioned with barcoded gel beads to generate single-cell gel bead-in-emulsion (GEM), and poly-adenylated transcripts were reverse-transcribed. Full-length cDNA and cell-barcode identifiers were PCR-amplified, and sequencing libraries were prepared and normalized to 3 nM for loading on a Novaseq 6000 (Illumina). Unsupervised clustering of cells from scRNA-seq was performed in R^30^ (version 4.2.1) using Seurat^31^ package (version 2.2) and Uniform Manifold Approximation and Projection (UMAP)^32^ R package (version 0.2.8.0) for dimensionality reduction. Visualization of single-cell transcriptomic data was done in R using RStudio^33^ and R packages tidyverse^34^ (version 1.3.1) and ggpubr^35^ (version 0.4.0).

#### Identification of marker genes and cell-type annotation.

Differential expression of every cluster was calculated using the ‘bimod’ test as implemented in the Seurat Find Markers function. Genes were found as marker genes with a log2 average differential expression of 0.25 and P < 0.05.

#### Macrophage subset identification and relative change calculation

Using our scRNA-seq data of CD14+ monocytes, we converted the population-averaged, log_2_ fold-change of the expression levels of the differentially expressed genes to fold-change level as follows:

$$y=\left\{\begin{array}{c} {2}^{x} \;\; for \;\; x>0 \\ \frac{1}{\left|{2}^{x}\right|} \;\;for \;\; x<0\end{array},\right.$$,

 where x is log_2_ fold-change and y is fold-change levels. Then, the data were used as an input for macrophage subset identification using MacSpectrum^14^ (https://macspectrum.uconn.edu). For relative change calculation, we obtained an averaged cell number of CD14+ monocyte subsets from patients (n = 3) and healthy control (n = 1) and calculate relative change as follows:

$$C=\frac{{x}_{2}-{x}_{1}}{{x}_{1}}$$,

where C is relative change, x_1_ is cell number of CD14+ monocyte subset from healthy control, and x_2_ is averaged cell number of CD14+ monocyte subset from patients.

#### Flow cytometry for investigate M1 monocyte population identified in the 10X experiments

To further investigate the M1 monocyte population identified in the 10X experiments, we used PBMCs from 13 patients (I-1, I-2, II-2, II-4, II-5, II-7, II-9, II-10, II-11, III-1, III-2, III-3 and III-4) and 5 healthy controls, stained with antibodies, and performed flow cytometry analysis. The antibodies included anti-CD14 PerCP/Cy5.5 (clone M5E2), anti- CD80 FITC (clone 2D10), anti-CD86 APC (clone IT2.2), anti-CD163 PE (clone GHI/61), and anti-CD206 PE-Cy7 (clone 15–2). All antibodies were purchased from BioLegend (San Diego, CA, USA). Cells were analyzed by LSRII flow cytometer (Becton Dickinson, USA). Gating strategy for M1 and M2 monocytes is depicted in Supplementary Fig. S5a. Data were processed by the FlowJo Software Version 10.8.1 (BD Life Sciences); website: https://www.flowjo.com/.

#### Single-cell RNA sequencing (scRNA-Seq) analysis of five additional PBMCs healthy individuals from publicly available data

The sorted five human PBMCs of healthy female donors aged 25–29 that supported the findings of this study were obtained from publicly available 10X Genomics datasets (Supplementary Table S5) (https://www.10xgenomics.com/resources/datasets)^36–40^, which included this published article Massively parallel digital transcriptional profiling of single cells^41^**.** The data were then included in our scRNA-seq dataset generated from our patients and a healthy control and re-analyzed using procedure described above. We noticed that cell topology and clusters changed after applying UMAP clustering^42^. To maintain cell-type classification consistency, we obtained a set of reference genes that described cell types in our initial scRNA-seq analysis and used it in cell-type identification by CIBERSORTx^16–18^. The difference in the number of monocyte subsets from patients and healthy controls was calculated as log_2_ fold-change value.

### Statistical analysis

Data were entered into GraphPad Prism (version 9.1.0) and analyzed using non-parametric tests (GraphPad Software, San Diego, CA, USA). For cell frequency and MFI from flow cytometry analysis, the results were shown in median of cell population. Kruskal–Wallis test (or non-parametric ANOVA) was used to evaluate flow cytometry data of LILRB1 surface expression. Manne-Whitney U test was used to evaluate flow cytometry data of other surface protein in cell subsets. Dunn test was used for multiple comparisons tests. P-value < 0.05 was considered statistically significant.

## Supplementary Information

Supplementary Information 1.

Supplementary Information 2.

Supplementary Information 3.

Supplementary Information 4.

Supplementary Information 5.

Supplementary Information 6.

Supplementary Information 7.

Supplementary Information 8.

Supplementary Information 9.

Supplementary Information 10.

## Data Availability

The datasets used and/or analyzed during the current study available from the corresponding author on reasonable request.
