# Supplementary material for: A LILRB1 variant with a decreased ability to phosphorylate SHP-1 leads to autoimmune diseases
Source: Sci Rep. 2022 Sep 14;12:15420. doi: 10.1038/s41598-022-19334-x (PMC9474825; doi:10.1038/s41598-022-19334-x)
Supplement: Supplementary file 1 — Supplementary Information 1. [file 41598_2022_19334_MOESM1_ESM.pdf]

a

	Recombination fraction (θ)							
	0	0.01	0.05	0.1	0.2	0.3	0.4	0.5
LOD	2.71	2.67	2.51	2.30	1.84	1.32	0.71	0

b

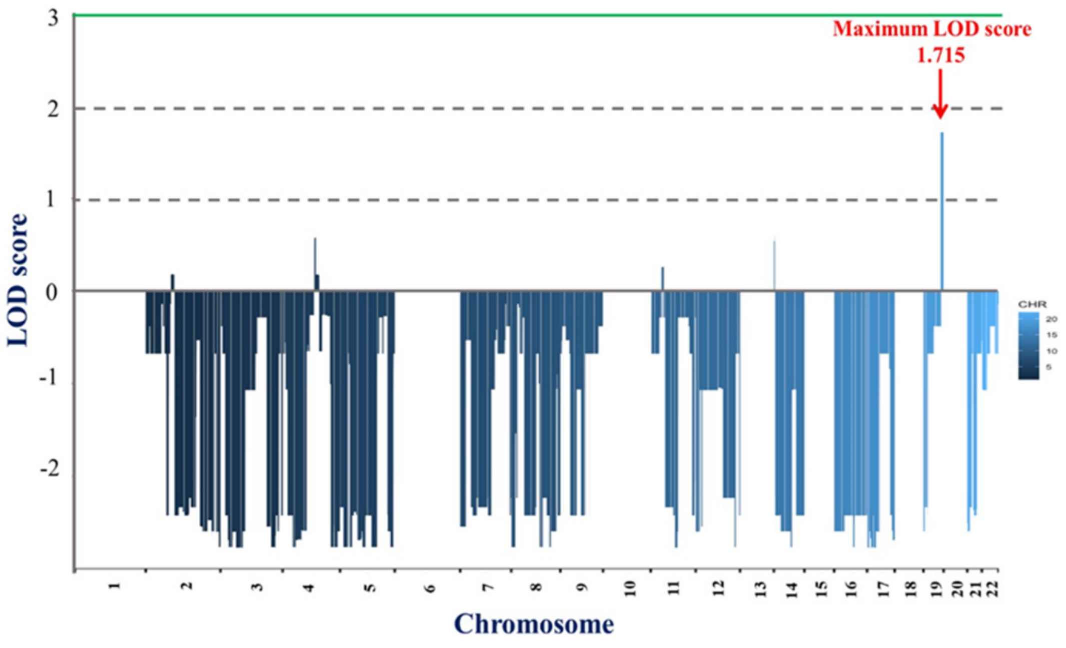

c

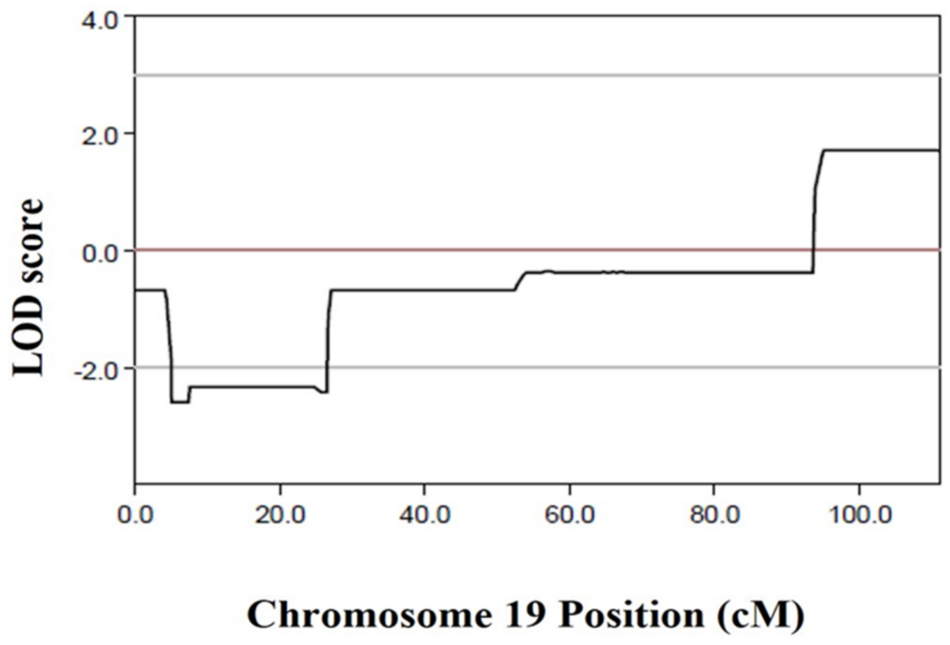

d

LILRB1

						p.G160					
Human	S	L	C	K	E	G	E	D	E	H	P
Rhesus	V	L	C	K	E	G	E	D	E	H	P
Mouse	I	P	I	K	A	G	P	Q	K	L	S
Dog	I	L	M	R	E	V	E	G	Q	P	S
Elephant	L	F	Y	K	E	G	E	H	K	R	S
Chicken	-	-	-	-	-	-	-	-	-	-	-
X_tropicalis	-	-	-	-	-	-	-	-	-	-	-
Zebrafish	-	-	-	-	-	-	-	-	-	-	-
Fruit fly	-	-	-	-	-	-	-	-	-	-	-
C. elegan	-	-	-	-	-	-	-	-	-	-	-

Based on the UCSC Comparative Genomics
(Vertebrate Multiz Alignment & Conservation)

Supplementary Figure S1. (a) Calculation LOD score by parametric linkage analysis (phase known data). **(b)** Whole genome linkage analysis performed by Merlin 1.1.2 software using an autosomal dominant model with the penetrance values being set at 0.6 revealed one region (arrows) on chromosome 19 with the maximum LOD score of 1.715. **(c)** A zoom-in of the linkage regions on chromosome 19. **(d)** Evolutionary conservation of amino acid across different species. The glycine residue at codon 160 is indicated in gray bar.
