# Supplementary material for: A LILRB1 variant with a decreased ability to phosphorylate SHP-1 leads to autoimmune diseases
Source: Sci Rep. 2022 Sep 14;12:15420. doi: 10.1038/s41598-022-19334-x (PMC9474825; doi:10.1038/s41598-022-19334-x)
Supplement: Supplementary file 2 — Supplementary Information 2. [file 41598_2022_19334_MOESM2_ESM.pdf]

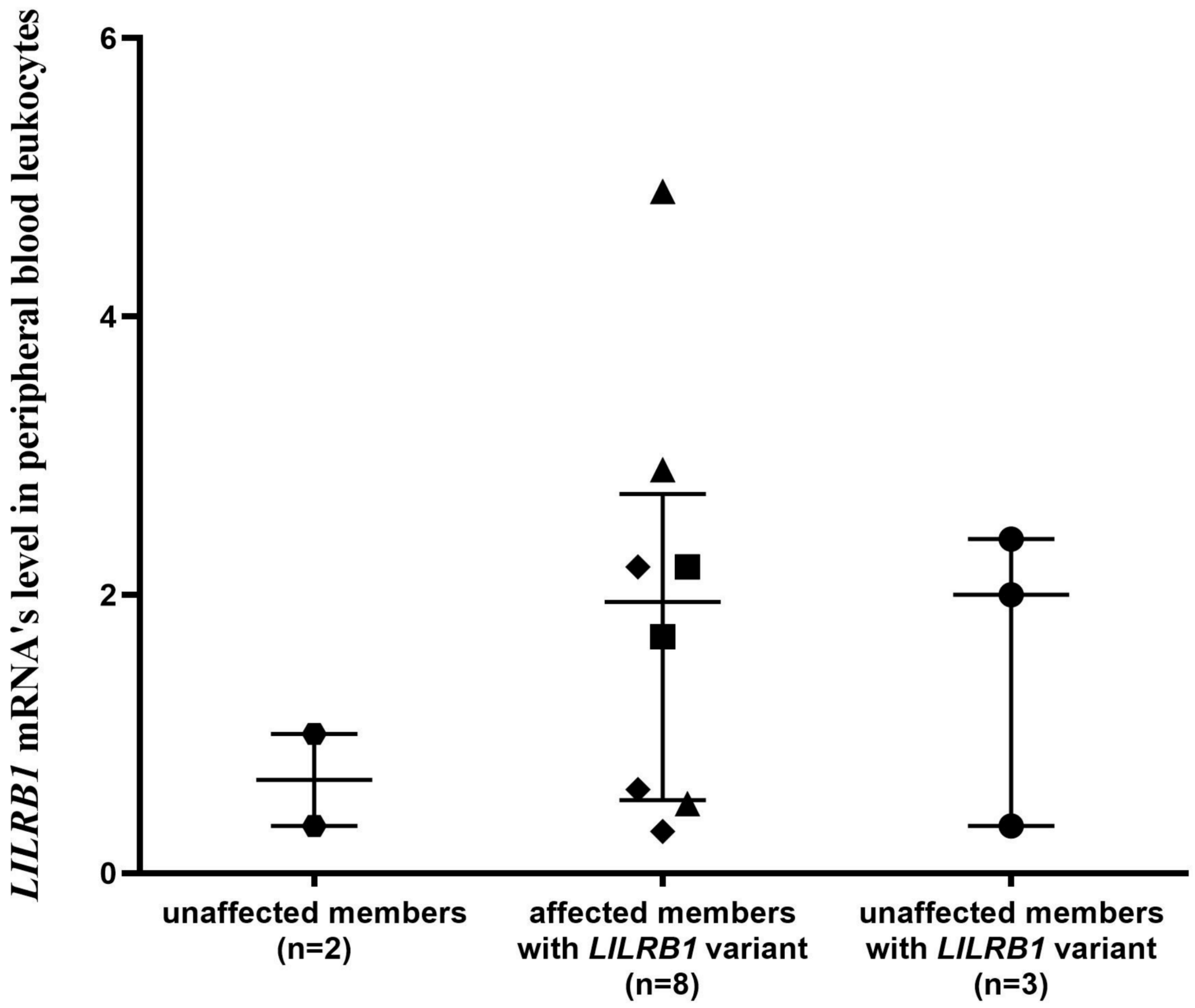

Supplementary Figure S2. The mRNA expression level of LILRB1 from this family detected by quantitative RT-PCR. Unaffected members (n = 2); affected members with *LILRB1* variant (n = 8); unaffected members with *LILRB1* variant (n = 3). The expression levels were calculated relative to a reference gene, *ACTB*. The data are presented as medians and interquartile ranges and were analyzed by the Kruskal-Wallis's test with Dunn's multiple test comparison.
