# Supplementary material for: A LILRB1 variant with a decreased ability to phosphorylate SHP-1 leads to autoimmune diseases
Source: Sci Rep. 2022 Sep 14;12:15420. doi: 10.1038/s41598-022-19334-x (PMC9474825; doi:10.1038/s41598-022-19334-x)
Supplement: Supplementary file 3 — Supplementary Information 3. [file 41598_2022_19334_MOESM3_ESM.pdf]

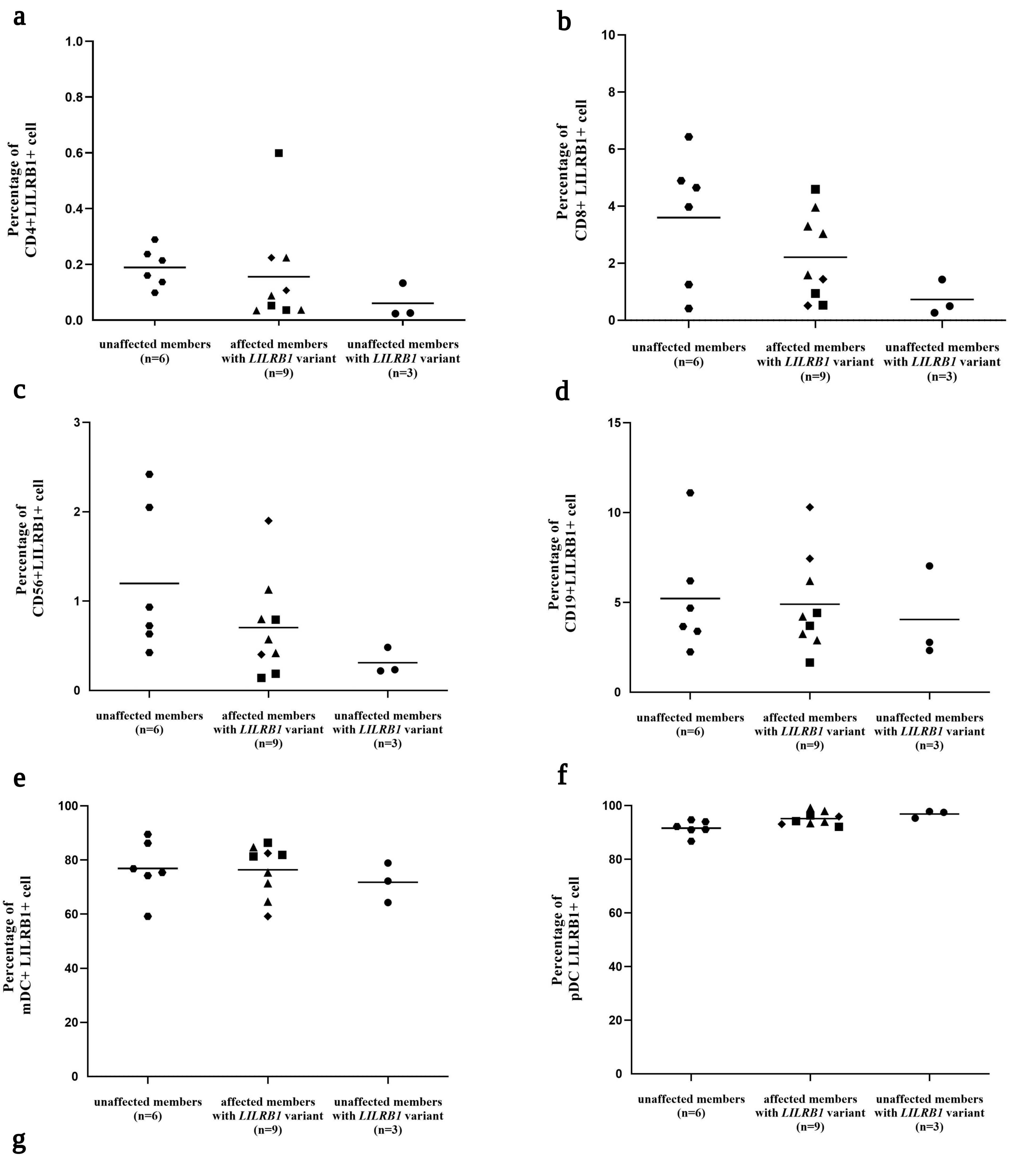

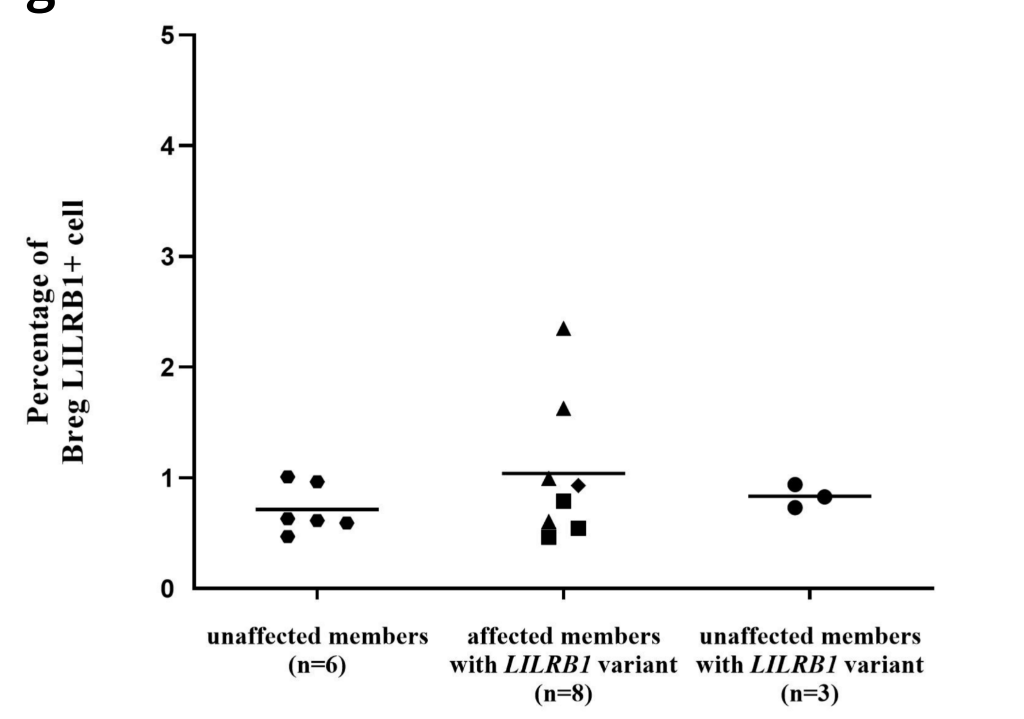

Supplementary Figure S3. Flow cytometry of the percentages of **(a)** CD4+ and **(b)** CD8+ T lymphocytes, **(c)** NK cells (CD16+CD56+), **(d)** B cells (CD19+), **(e)** myeloid dendritic cells (mDCs; CD1c+CD11c+), **(f)** plasmacytoid dendritic cells (pDCs; CD123+CD303+) and **(g)** regulatory B lymphocytes (Breg;CD71+CD73-CD25+CD19+). Horizontal bar indicates mean of the percentage of cell type in each group.
