# Supplementary material for: A LILRB1 variant with a decreased ability to phosphorylate SHP-1 leads to autoimmune diseases
Source: Sci Rep. 2022 Sep 14;12:15420. doi: 10.1038/s41598-022-19334-x (PMC9474825; doi:10.1038/s41598-022-19334-x)
Supplement: Supplementary file 4 — Supplementary Information 4. [file 41598_2022_19334_MOESM4_ESM.pdf]

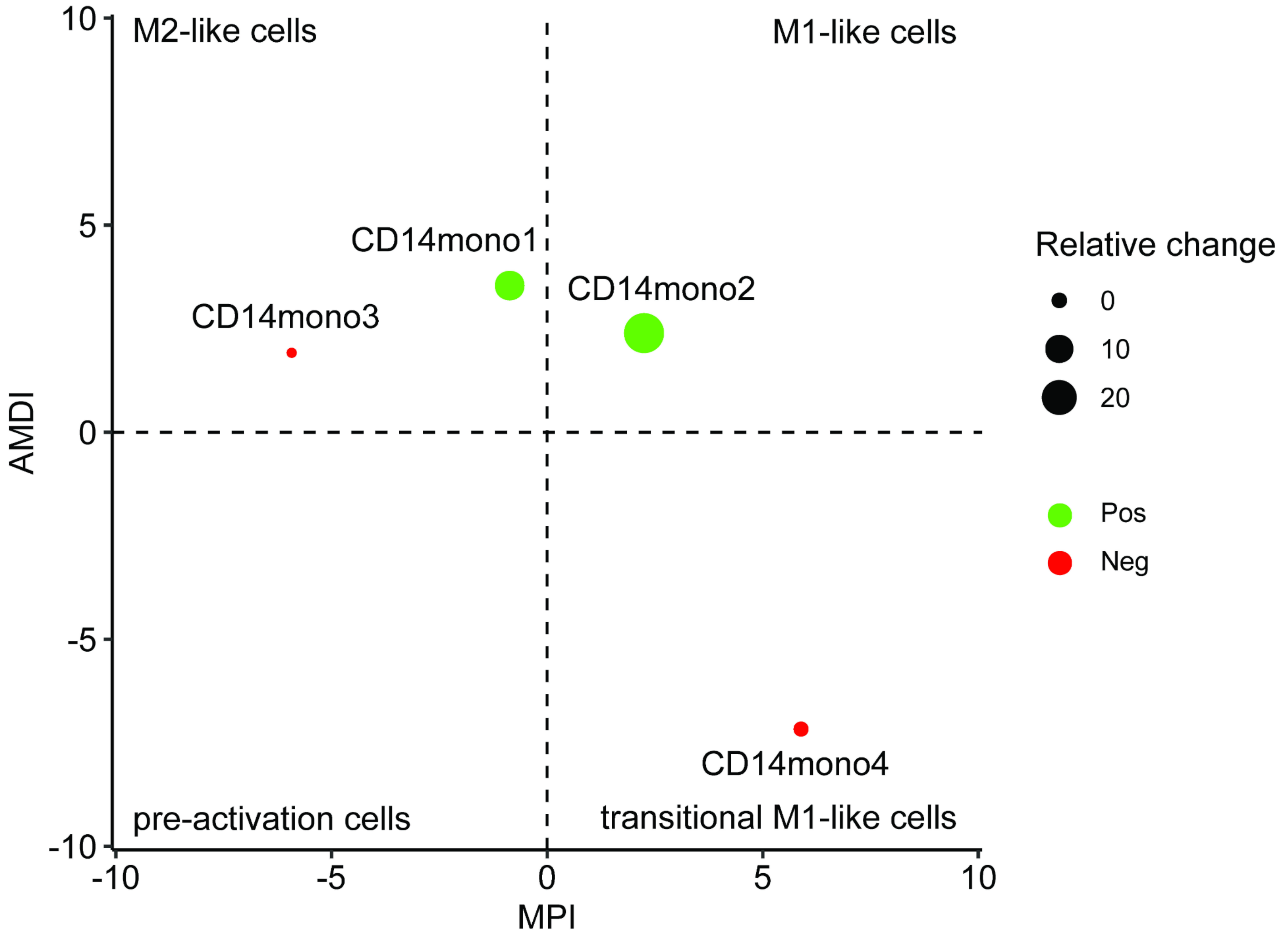

Supplementary Figure S4. Macrophage subset characterization and relative change of CD14+ monocyte subsets from patients with LILRB1 variant. CD14+ monocytes on the 2-index (macrophage polarization index; MPI, and activation-induced macrophage differentiation index; AMDI) MacSpectrum plot were designated as “M2-like”, “M1-like”, “transitional M1-like”, and “preactivation” cell types. Point size indicates relative change of cell number of specific CD14+ monocyte subset in patients compared to healthy control. Green and red colors represent positive and negative change, respectively.
