# Supplementary material for: A LILRB1 variant with a decreased ability to phosphorylate SHP-1 leads to autoimmune diseases
Source: Sci Rep. 2022 Sep 14;12:15420. doi: 10.1038/s41598-022-19334-x (PMC9474825; doi:10.1038/s41598-022-19334-x)
Supplement: Supplementary file 5 — Supplementary Information 5. [file 41598_2022_19334_MOESM5_ESM.pdf]

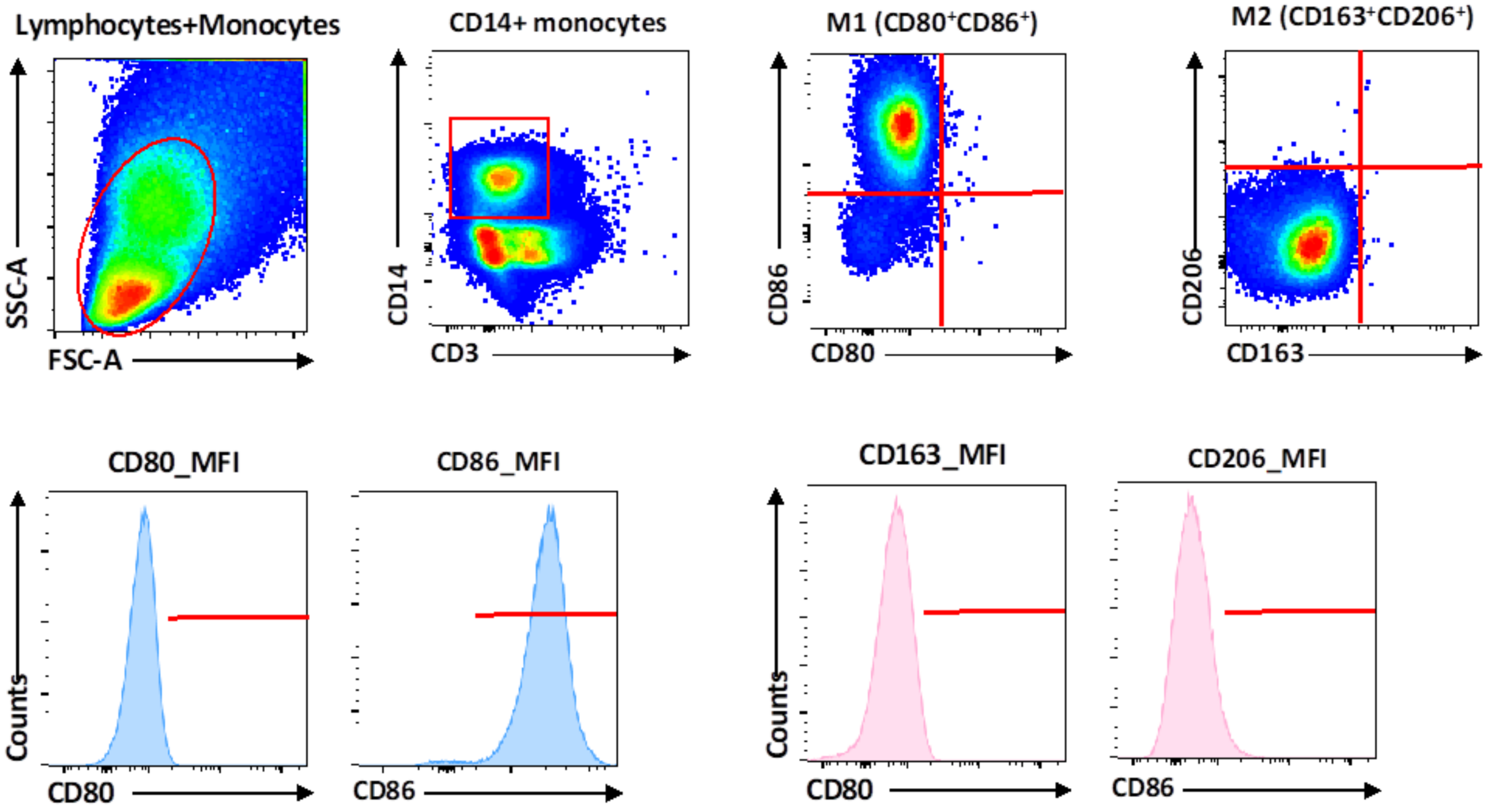

Supplementary Fig S5 . Gating strategy of M1 and M2 monocytes. Data were processed by the FlowJo Software Version 10.8.1 (BD Life Sciences); website: https://www.flowjo.com/.
