# Supplementary material for: A LILRB1 variant with a decreased ability to phosphorylate SHP-1 leads to autoimmune diseases
Source: Sci Rep. 2022 Sep 14;12:15420. doi: 10.1038/s41598-022-19334-x (PMC9474825; doi:10.1038/s41598-022-19334-x)
Supplement: Supplementary file 6 — Supplementary Information 6. [file 41598_2022_19334_MOESM6_ESM.pdf]

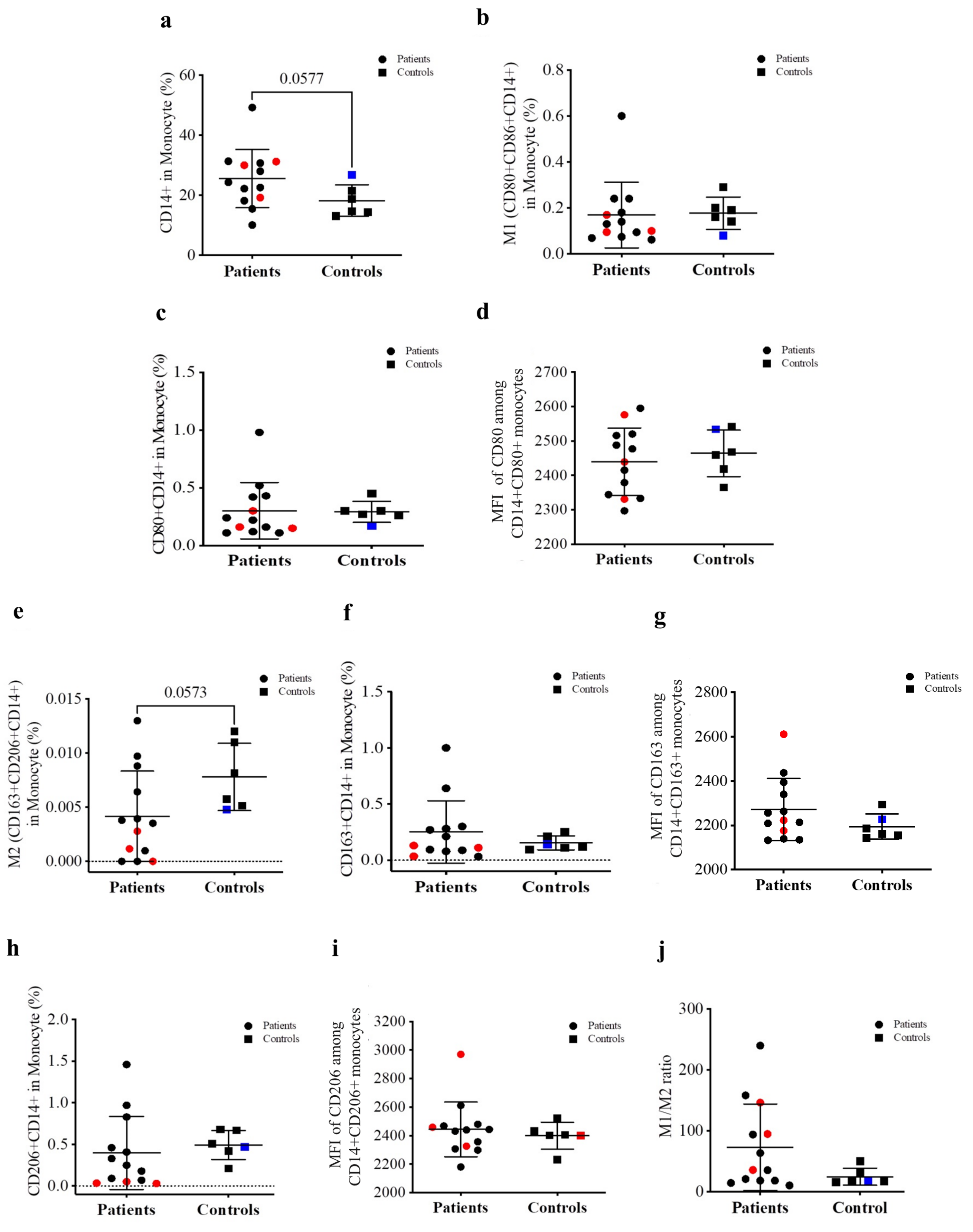

Supplementary Figure S6. Flow cytometry results of the frequency and mean fluorescence intensity (MFI) of different cell types from PBMCs of patients and controls. **(a)** frequency of CD14+ monocytes. **(b)** frequency of M1 (CD14+C80+CD86+) monocytes. **(c)** frequency of CD14+CD80+ monocytes. **(d)** MFI of CD80 in CD14+CD80+ monocytes. **(e)** frequency of M2 (CD14+CD163+CD206+) monocytes. **(f)** frequency of CD14+CD163+ monocytes. **(g)** MFI of CD163 in CD14+CD163+ monocytes. **(h)** frequency of CD14+CD206+ monocytes. **(i)** MFI of CD206 in CD14+CD206+ monocytes. **(j)** ratio of M1/M2 cells. Red and blue dots indicate samples of patients and controls that were concurrently investigated in scRNA-seq experiment, respectively.
