# Supplementary material for: A LILRB1 variant with a decreased ability to phosphorylate SHP-1 leads to autoimmune diseases
Source: Sci Rep. 2022 Sep 14;12:15420. doi: 10.1038/s41598-022-19334-x (PMC9474825; doi:10.1038/s41598-022-19334-x)
Supplement: Supplementary file 7 — Supplementary Information 7. [file 41598_2022_19334_MOESM7_ESM.pdf]

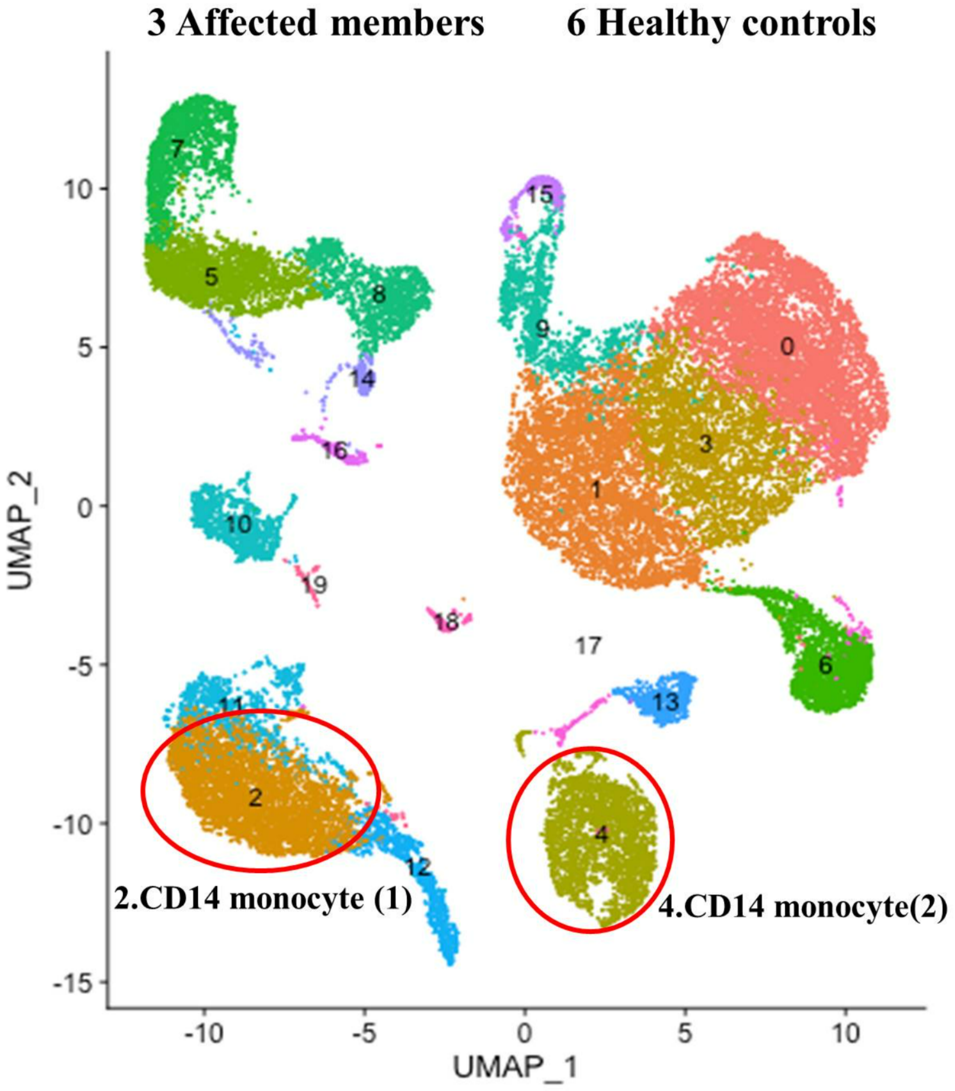

Supplementary Figure S7. UMAP showed the topology of cells of the nine samples (three patients, one Thai healthy control, and five publicly available healthy controls). Visualization of single-cell transcriptome data was done in R (v. 4.2.1; https://www.R-project.org) using RStudio (https://www.rstudio.com) and R packages tidyverse (v. 1.3.1; https://doi.org/10.21105/joss.01686) and ggpubr (v. 0.4.0; https://CRAN.R-project.org/package=ggpubr).
