# Supplementary material for: A LILRB1 variant with a decreased ability to phosphorylate SHP-1 leads to autoimmune diseases
Source: Sci Rep. 2022 Sep 14;12:15420. doi: 10.1038/s41598-022-19334-x (PMC9474825; doi:10.1038/s41598-022-19334-x)
Supplement: Supplementary file 8 — Supplementary Information 8. [file 41598_2022_19334_MOESM8_ESM.pdf]

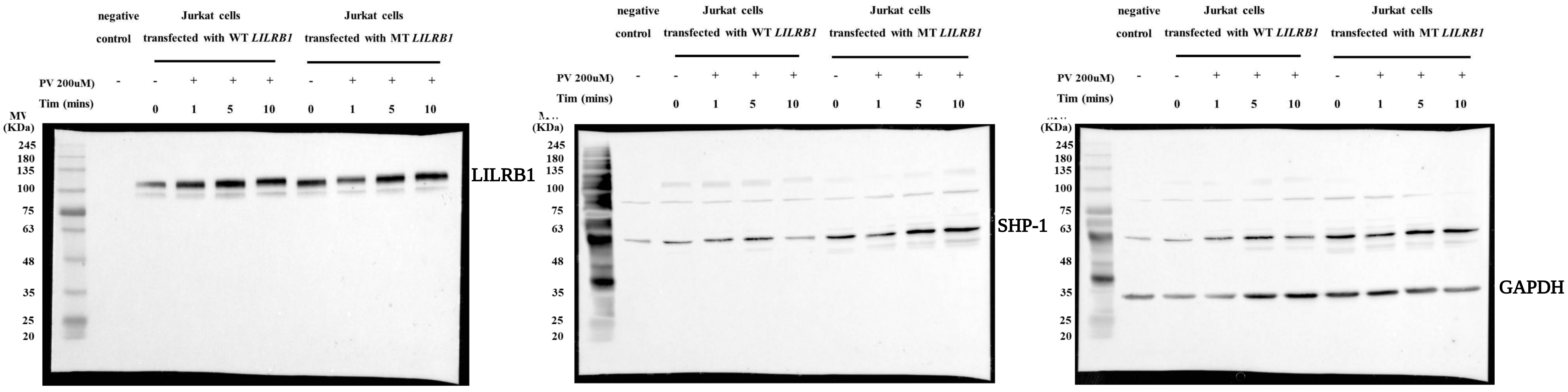

Supplementary Figure S8. Uncropped images of Western blots presented in Fig 2a. The protein levels of LILRB1 and SHP-1 in the Jurkat cells transfected with the wild-type (WT) *LILRB1* and mutant (MT) *LILRB1* in different time points as detected by Western blotting. GAPDH was used as a loading control. Control denotes Jurkat cells without transfection and PV denotes pervanadate.
