# Supplementary material for: A LILRB1 variant with a decreased ability to phosphorylate SHP-1 leads to autoimmune diseases
Source: Sci Rep. 2022 Sep 14;12:15420. doi: 10.1038/s41598-022-19334-x (PMC9474825; doi:10.1038/s41598-022-19334-x)
Supplement: Supplementary file 9 — Supplementary Information 9. [file 41598_2022_19334_MOESM9_ESM.pdf]

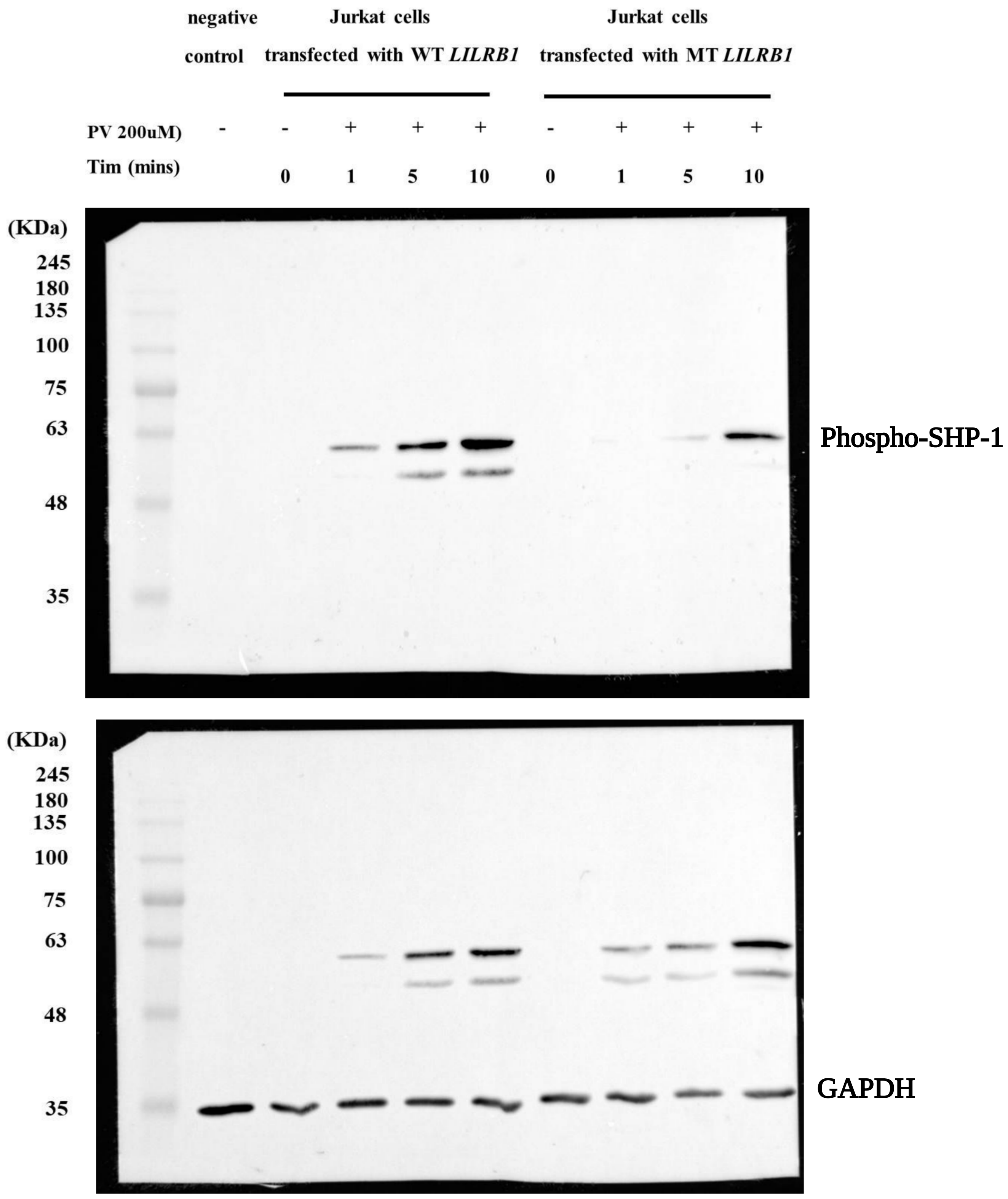

Supplementary Figure S9. Uncropped images of Western blots presented in Fig. 2c. The tyrosine phosphorylation status of SHP-1 was examined using Western blot analysis in the Jurkat cells transfected with the wild-type (WT) *LILRB1* and mutant (MT) *LILRB1* in different time points.GAPDH was used as a loading control. Control denotes Jurkat cells without transfection and PV denotes pervanadate
