# Supplementary material for: A LILRB1 variant with a decreased ability to phosphorylate SHP-1 leads to autoimmune diseases
Source: Sci Rep. 2022 Sep 14;12:15420. doi: 10.1038/s41598-022-19334-x (PMC9474825; doi:10.1038/s41598-022-19334-x)
Supplement: Supplementary file 10 — Supplementary Information 10. [file 41598_2022_19334_MOESM10_ESM.docx]

**Supplementary Information**

**A LILRB1 variant with a decreased ability to phosphorylate SHP-1 leads to autoimmune diseases**

Thivaratana Sinthuwiwat^1,2,3,4^, Supranee Buranapraditkun^5,6,7^, Wuttichart Kamolvisit^2,3^, Siraprapa Tongkobpetch^2,3^, Wanna Chetruengchai^1,2,3^, Chalurmpon Srichomthong^2,3^, Adjima Assawapitaksakul^2,3^, Chureerat Phokaew^2,3,8^, Patipark Kueanjinda ^9,10^, Tanapat Palaga^10,11^, Tadech Boonpiyathad^12^, Kanya Suphapeetiporn^2,3^, Nattiya Hirankarn^9,10^, and Vorasuk Shotelersuk^2,3,*^

^1^Interdisciplinary Program of Biomedical Sciences, Graduate School, Chulalongkorn University, Bangkok, Thailand

^2^Center of Excellence for Medical Genomics, Medical Genomics Cluster, Department of Pediatrics, Faculty of Medicine, Chulalongkorn University, Bangkok 10330, Thailand

^3^Excellence Center for Genomics and Precision Medicine, King Chulalongkorn Memorial Hospital, the Thai Red Cross Society, Bangkok, Thailand

^4^Division of Cytogenetics, Chulabhorn Learning and Research Centre, Chulabhorn Royal Academy, Bangkok, Thailand^5^Division of Allergy and Clinical Immunology, Department of Medicine, King Chulalongkorn Memorial Hospital, Faculty of Medicine, Chulalongkorn University, Thai Red Cross Society, Bangkok 10330, Thailand^6^Center of Excellence in Vaccine Research and Development (Chula Vaccine Research Center- Chula VRC), Faculty of Medicine, Chulalongkorn University, Bangkok 10330, Thailand^7^Thai Pediatric Gastroenterology, Hepatology and Immunology (TPGHAI) Research Unit, Faculty of Medicine, King Chulalongkorn Memorial Hospital, Chulalongkorn University, The Thai Red Cross Society, Bangkok 10330, Thailand^8^Research Affairs, Faculty of Medicine, Chulalongkorn University, Bangkok 10330, Thailand

^9^Department of Microbiology, Faculty of Medicine, Chulalongkorn University, Bangkok,

Thailand

^10^Center of Excellence in Immunology and Immune-mediated Diseases, Chulalongkorn University, Bangkok, Thailand

^11^Department of Microbiology, Faculty of Science, Chulalongkorn University, Bangkok, Thailand

^12^Allergy and Clinical Immunology, Department of Medicine, Phramongkutklao Hospital, Bangkok, Thailand

**Supplementary Figure Legends**

**Supplementary Figure S1.** (**a**) Calculation LOD score by parametric linkage analysis (phase known data). (**b**) Whole genome linkage analysis performed by Merlin 1.1.2 software using an autosomal dominant model with the penetrance values being set at 0.6 revealed one region (arrows) on chromosome 19 with the maximum LOD score of 1.715. (**c**) A zoom-in of the linkage regions on the chromosome 19. (**d**) Evolutionary conservation of amino acid across different species. The glycine residue at codon 160 is indicated in gray bar.

**Supplementary Figure S2.** The mRNA expression level of *LILRB1* from this family detected by quantitative RT-PCR. Unaffected members (n = 2). Affected members with *LILRB1* variant (n = 8). Unaffected members with *LILRB1* variant (n = 3). The expression levels were calculated relative to a reference gene, *ACTB*. The data are presented as medians and interquartile ranges and were analyzed by the Kruskal-Wallis’s test with Dunn’s multiple test comparison.

**Supplementary Figure S3.** Flow cytometry of the percentages of (**a**) CD4+ and (**b**) CD8+ T lymphocytes, (**c**) NK cells (CD16+CD56+), (**d**) B cells (CD19+), (**e**) myeloid dendritic cells (mDCs; CD1c+CD11c+), (**f**) plasmacytoid dendritic cells (pDCs; CD123+CD303+) and (**g**) regulatory B lymphocytes (Breg; CD71+CD73-CD25+CD19+). Horizontal bar indicates mean of the percentage of cell type in each group.

**Supplementary Figure S4.** Macrophage subset characterization and relative change of CD14+ monocyte subsets from patients with LILRB1 variant. CD14+ monocytes on the 2-index (macrophage polarization index; MPI, and activation-induced macrophage differentiation index; AMDI) MacSpectrum plot were designated as “M2-like”, “M1-like”, “transitional M1-like”, and “preactivation” cell types. Point size indicates relative change of cell number of specific CD14+ monocyte subset in patients compared to healthy control. Green and red colors represent positive and negative change, respectively.

**Supplementary Figure S5.** Gating strategy of M1 and M2 monocytes. Data were processed by the FlowJo Software Version 10.8.1 (BD Life Sciences); website: https://www.flowjo.com/.

**Supplementary Figure S6.** Flow cytometry results of the frequency and mean fluorescence intensity (MFI) different cell types from PBMCs of patients and controls. (**a**) frequency of CD14+ monocytes. (**b**) frequency of M1 (CD14+C80+CD86+) monocytes. (**c**) frequency of CD14+CD80+ monocytes. (**d**) MFI of CD80 in CD14+CD80+ monocytes. (**e**) frequency of M2 (CD14+CD163+CD206+) monocytes. (**f**) frequency of CD14+CD163+ monocytes. (**g**) MFI of CD163 in CD14+CD163+ monocytes. (**h**) frequency of CD14+CD206+ monocytes. (**i**) MFI of CD206 in CD14+CD206+ monocytes. (**j**) ratio of M1/M2 cells. Red and blue dots indicate samples of patients and controls that were concurrently investigated in scRNA-seq experiment, respectively.

**Supplementary Figure S7.** UMAP showed the topology of cells of the nine samples (three patients, one Thai healthy control, and five publicly available healthy controls). Visualization of single-cell transcriptome data was done in R (v. 4.2.1; https://www.R-project.org) using RStudio (http://www.rstudio.com) and R packages tidyverse (v. 1.3.1; https://doi.org/10.21105/joss.01686) and ggpubr (v. 0.4.0; https://CRAN.R-project.org/package=ggpubr).

**Supplementary Figure S8.** Uncropped images of Western blots presented in Fig. 2a. The protein levels of LILRB1 and SHP-1 in Jurkat cells transfected with the wild-type (WT) or mutant (MT) LILRB1 and treated with pervanadate (PV) at different time points. GAPDH was used as a loading control. Control denotes Jurkat cells without any plasmid transfection. PV denotes pervanadate.

**Supplementary Figure S9.** Uncropped images of Western blots presented in Fig. 2c. The tyrosine phosphorylation status of SHP-1 was examined in Jurkat cells transfected with the wild-type (WT) or mutant (MT) LILRB1 and treated with pervanadate (PV) at different time points. GAPDH was used as a loading control. Control denotes Jurkat cells without any plasmid transfection. PV denotes pervanadate.

**Supplementary Table Legends**

**Supplementary Table S1.** Clinical characteristics and laboratory findings of the nine patients with autoimmune diseases and the LILRB1 variant.

**Supplementary Table S2.** Filtering criteria for the analysis of whole exome sequencing of the nine patients.

**Supplementary Table S3.** The three non-synonymous exonic variants with allele frequencies <1% in public and the in-house databases and present in all 9 patients.

**Supplementary Table S4.** Frequencies of cells from single cell RNA sequencing (scRNA-seq) of three patients and a healthy sex-, age- and ethnic-matched control.

**Supplementary Table S5.** Details of the nine samples composed of three patients, one Thai healthy control and five healthy controls.

**Supplementary Table S6.** Marker and frequencies of cells from single-cell RNA sequencing (scRNA-seq) of three patients and six healthy sex-matched controls.

**Supplementary Table S7.** Log_2_FC of monocyte and M1 cells compared between three patients and six healthy sex-matched controls.

**Supplementary Table S8.** Primers for PCR amplification and sequencing.

**Supplementary Table S1.** Clinical characteristics and laboratory findings of the nine patients with autoimmune diseases and the *LILRB1* variant.

|  | I-2 | II-1 | II-3 | II-5 | II-7 | II-9 | III-2 | III-3 | III-4 |
| --- | --- | --- | --- | --- | --- | --- | --- | --- | --- |
| Sex | F | F | F | F | M | F | M | F | F |
| Age (y) | 70 | 52 | 50 | 47 | 45 | 42 | 25 | 23 | 19 |
| Age at onset (y) | 67 | 48 | 42 | 42 | - | 33 | 21 | 16 | 16 |
| Diagnosis | Hashimoto’s thyroiditis | SLE | Hashimoto’s thyroiditis | Graves’  disease | Graves’ disease | Hashimoto’s thyroiditis | Graves’  disease | Graves’ disease | SLE |
| Clinical manifestations | Cold intolerance | Severe multiple non-migratory joint pain | Painless Goiter | Significant weight loss, Palpitation | Palpitation | Lethargy | Palpitation | Exophthalmos, Significant weight loss, Palpitation | Malar rash, Lupus nephritis  class V, thrombocytopenia |
| ANA | N/A | Positive | N/A | N/A | N/A | N/A | N/A | N/A | N/A |
| Anti-dsDNA | N/A | N/A | N/A | N/A | N/A | N/A | N/A | N/A | N/A |
| Anti-Sm | N/A | Positive | N/A | N/A | N/A | N/A | N/A | N/A | N/A |
| Anti  phospholipid antibody | N/A | N/A | N/A | N/A | N/A | N/A | N/A | N/A | Positive |
| Anti  cardiolipin IgG | N/A | N/A | N/A | N/A | N/A | N/A | N/A | N/A | Positive |
| C4 | N/A | Low | N/A | N/A | N/A | N/A | N/A | N/A | N/A |
| FT4  (0.8-1.8 ng/mL) | 1.11 | N/A | 1.18 | 1.77 | 1.28 | 0.74 | 2.14 | 3.23 | N/A |
| TSH  (0.3-4.1 uIU/mL) | 7.8 | N/A | 4.24 | 0.006 | 0.266 | 25 | 0.01 | 0.003 | N/A |
| Anti-thyroglobulin  (<4.11 IU/mL) | N/A | N/A | >1,000 | N/A | N/A | N/A | N/A | N/A | N/A |
| Anti-thyroid peroxidase  (<5.61 IU/mL) | 452.3 | N/A | >1,000 | N/A | N/A | N/A | N/A | N/A | N/A |
| CRP-QT  (<3 mg/L) | N/A | 44.800 | N/A | N/A | N/A | N/A | N/A | N/A | N/A |
| Medication | Eltroxin | Imuprin  Prednisolone  Hydroquin | Eltroxin | Methimazole | Eltroxin  after radioactive iodine therapy | Eltroxin | Propylthiouracil, Methimazole | Methimazole | Chloroquine Tacrolimus |

**Supplementary Table S2.** Filtering criteria for the analysis of whole exome sequencing of the nine patients.

| **Filtering criteria:** | **Number of variants** |
| --- | --- |
| 1.in any of the 9 patients | 215,364 |
| 2.in all 9 patients | 2,518 |
| 3.in or close to the coding regions and not synonymous | 571 |
| 4.with coverage >10x; with minor allele frequency <1% in the 1000 Genomes Project and gnomAD; and being non-synonymous exonic variants | 11 |
| 5. prediction software; deleterious, damaging or possibly damage | 3 |
| 6.located in the identified linked region on chromosome 19q13.4 | 1 |
| Gene: *LILRB1* |  |
| Coordinate: chr19:55143506 |  |
| Genotype: Heterozygous |  |
| Variant: c.479G>A |  |
| Protein: p. Gly160Glu |  |
| SIFT: deleterious (0.01) |  |
| PolyPhen-2: Possibly damage (0.658) |  |

**Supplementary Table S3.** The three non-synonymous exonic variants with allele frequencies <1% in public and the in-house databases and present in all 9 patients.

| Gene | Chromosome | Genetic  coordinate | Amino acid change | Prediction software | Cell or tissue expression | Associated diseases |
| --- | --- | --- | --- | --- | --- | --- |
| *LILRB1* | 19q13.42 | 55143506 | p.G160E | Damaging | T cells, B cells, dendritic cells, NK cells,  macrophages and monocytes | Hashimoto’s thyroiditis, Graves’ disease and SLE |
| *OTOP1* | 4p16.3 | 4190576 | p.R598P | Damaging | Testis, skin and esophagus | - |
| *FRG1* | 4q35.2 | 190876293 | p.P140Q | Damaging | All tissue types | Facioscapulohumeral muscular dystrophy-1 |

**Supplementary Table S4.** Frequencies of cells from single cell RNA sequencing (scRNA-seq) of three patients and a healthy sex-, age- and ethnic-matched control.

| Cluster | Cell numbers  in the three patients combined | Cell numbers  in the healthy control | Log_2_FC* |
| --- | --- | --- | --- |
| 0.CD8+ T cell | 3769 | 964 | 0.4 |
| 1.NK cell | 3569 | 917 | 0.4 |
| 2.CD14+ monocyte (1) | 3731 | 97 | 3.7^#^ |
| 3.B cell (1) | 2235 | 1458 | -1.0 |
| 4.CD14+monocyte (2) | 3191 | 35 | 4.9^#^ |
| 5.CD14+monocyte (3) | 104 | 3039 | -6.5^#^ |
| 6.Naïve CD4+ T cell (1) | 543 | 2451 | -1.8 |
| 7.Naïve CD4+ T cell (2) | 1714 | 114 |  |
| 8.FCGR3A+ monocyte | 1352 | 434 | 0.1 |
| 9.CD4+ T cell | 999 | 563 | -0.8 |
| 10.Basophil | 733 | 562 | -1.2 |
| 11.CD1C- CD141- dendritic cell | 852 | 357 | -0.3 |
| 12.CD14+monocyte (4) | 769 | 316 | -0.3 |
| 13.platelet (1) | 519 | 143 | 0.3 |
| 14.myeloid/conventional dendritic cells 2 | 273 | 356 | -2.0 |
| 15.platelet (2) | 422 | 163 | -0.2 |
| 16.plasmacytoid dendritic cell | 194 | 183 | -1.5 |
| 17.Memory B cell | 156 | 112 | -1.1 |
| 18.B cell (2) | 142 | 116 | -1.3 |
| 19.Treg | 100 | 63 | -0.9 |

Log_2_FC* denotes log 2 of fold changes.

# denotes clusters which Log_2_FC > 2 or < -2

**Supplementary Table S5.** Details of the nine samples composed of three patients, one Thai healthy control and five healthy controls.

| Sample name | Estimated  Number of Cells | Sample  type | Tissue  type | Library | Cell ranger | Sequencer | Reference |
| --- | --- | --- | --- | --- | --- | --- | --- |
| G6250 | 8,990 | disease | PBMC | the Chromium Single Cell 5′ library preparation kit | v 3.1.0 | Novaseq 6000 |  |
| G6255 | 8,285 |  |  |  |  |  |  |
| G6260 | 9,811 |  |  |  |  |  |  |
| G2000046 (H7) | 12,673 | control |  |  |  |  |  |
| H1  (age 25) | 10,194 | Control from public data databases | Human PBMCs | The Chromium Next GEM Single Cell 3' Reagent Kits v3.1 (Dual Index) | 4.0.0 |  | 38 |
| H2  (age 25-30) | 16,000 |  | 10k Human PBMCs | The Chromium Single Cell 3' Reagent Kits User Guide (v3.1 Chemistry Dual Index) | 6.1.0 |  | 39 |
| H3  (age 25-30) |  |  | 10k  Human PBMCs | The Chromium Single Cell 5' Reagent Kits User Guide (v2 Chemistry Dual Index) |  |  | 40 |
| H5  (age 25-30) |  |  | 10k Human PBMCs | The Chromium Single Cell 5' Reagent Kits User Guide (v2 Chemistry Dual Index) |  |  | 41 |
| H6  (age 25-30) | 33,000 |  | 20k Human PBMCs | The Chromium Single Cell 5’ Reagent Kits User Guide (v2 Chemistry Dual Index) |  |  | 42 |

^38^ *PBMCs from a Healthy Donor: Whole Transcriptome Analysis,* Single Cell Gene Expression Dataset by Cell Ranger 4.0.0. https://www.10xgenomics.com/resources/datasets/pbm-cs-from-a-healthy-donor-whole-transcriptome-analysis-3-1-standard-4-0-0. Accessed 1 June 2022.

# ^39^ *10K PBMCs from human,3’ (v3.1)* *Chromium X*, Single Cell Gene Expression Dataset by Cell Ranger 6.1.0.

# https://www.10xgenomics.com/resources/datasets/10k-human-pbmcs-3-ht-v3-1-chromium-x-3-1-high. Accessed 1 June 2022.

^40^ *PBMCs from human,5’ (v2.0)* *Chromium controller*, Single Cell Immune Profiling Dataset by Cell Ranger 6.1.0. https://www.10xgenomics.com/resources/datasets/10-k-human-pbm-cs-5-v-2-0-chromium-controller-2-standard-6-1-0. (Accessed 1 June 2022

# ^41^*10K PBMCs from human,5’ (v2.0)* *Chromium X*, Single Cell Immune Profiling Dataset by Cell Ranger 6.1.0. https://www.10xgenomics.com/resources/datasets/10-k-human-pbm-cs-5-v-2-0-chromium-x-2-standard-6-1-0. Accessed 1 June 2022.

^42^ *20K PBMCs from human,5’ HT (v2.0)*, Single Cell Immune Profiling Dataset by Cell Ranger 6.1.0. https://www.10xgenomics.com/resources/datasets/20-k-human-pbm-cs-5-ht-v-2-0-2-high-6-1-0. Accessed 1 June 2022.

**Supplementary Table S6.** Marker and frequencies of cells from single-cell RNA sequencing (scRNA-seq) of three patients and six healthy sex-matched controls.

| cluster | Marker | cell type | Cell numbers in the 3 patients combined | Cell numbers in the 6 healthy controls |
| --- | --- | --- | --- | --- |
| 0 | IL7R,SELL,TRC7,CCR7 | naïve CD4+ T cell | 0 | 7129 |
| 1 | IL7R | CD4+ T cell | 0 | 5182 |
| 2 | CD14,LYZ | CD14+ monocyte (1) | 4740 | 0 |
| 3 | IL7R | CD4+ T cell | 0 | 3645 |
| 4 | CD14,LYZ | CD14+ monocyte (2) | 0 | 3216 |
| 5 | CD8A | CD8+ T cell | 2962 | 0 |
| 6 | CD3G | T cell | 0 | 2548 |
| 7 | NKG7,GNLY | NK cell | 1987 | 0 |
| 8 | IL7R | CD4+ T cell | 1657 | 0 |
| 9 | CD8A | CD8+ T cell | 0 | 1545 |
| 10 | MS4A1,CD19 | B cell | 1267 | 0 |
| 11 | LYZ | monocyte | 1154 | 0 |
| 12 | FCGR3A,MS4A7,LYZ | CD16+ monocyte | 1150 | 0 |
| 13 | MS4A1,CD19,IGHM,IGHD | naïve B cell | 0 | 795 |
| 14 | CCR7 | Naïve CD4+ T cell (1) | 416 | 0 |
| 15 | NKG7,GNLY | NK cell | 0 | 374 |
| 16 | PPBP | platelet (1) | 306 | 0 |
| 17 | unknown | | 0 | 202 |
| 18 | PPBP | platelet (2) | 0 | 181 |
| 19 | HLA-DRA | Dendritic cell | 105 | 0 |

**Supplementary Table S7.** Log_2_FC of monocyte and M1 cells compared between three patients and six healthy sex-matched controls.

|  | Marker | Cluster | Affected (average) | Healthy (average) | Log_2_FC* |
| --- | --- | --- | --- | --- | --- |
| Monocytes | CD14 | 2,4 | 1580 | 536 | 1.56 |
| M1 | LYZ | 2,4 | 1580 | 536 | 1.56 |
| M2 | CLEC10A | - | - | - | - |

Log_2_FC* denotes log 2 of fold changes

# denotes clusters which Log_2_FC > 2 or < -2

**Supplementary Table S8.** Primers for PCR amplification and sequencing.

| **Primer sequence (5’-3’)** | **Sequences** |
| --- | --- |
| LILRB1-long1F | CTGCTCATGACATTGATGCTCTG |
| LILRB1-long1R | CGCTACCATAGTAACAGCGATAC |
| LILRB1-long2F | GTATCGCTGTTACTATGGTAGCG |
| LILRB1-long2R | ATTACAGGCACTGCCACCACAT |
| LILRB1specificF | GTATCGCTGTTACTATGGTAGCG |
| LILRB1-1R | GAATTTCTCACCTAGGACCAGGA |
